# Misty Mountain clustering: application to fast unsupervised flow cytometry gating

**DOI:** 10.1186/1471-2105-11-502

**Published:** 2010-10-09

**Authors:** István P Sugár, Stuart C Sealfon

**Affiliations:** 1 Department of Neurology and Center for Translational Systems Biology, Mount Sinai School of Medicine, New York, NY, USA

## Abstract

**Background:**

There are many important clustering questions in computational biology for which no satisfactory method exists. Automated clustering algorithms, when applied to large, multidimensional datasets, such as flow cytometry data, prove unsatisfactory in terms of speed, problems with local minima or cluster shape bias. Model-based approaches are restricted by the assumptions of the fitting functions. Furthermore, model based clustering requires serial clustering for all cluster numbers within a user defined interval. The final cluster number is then selected by various criteria. These supervised serial clustering methods are time consuming and frequently different criteria result in different optimal cluster numbers. Various unsupervised heuristic approaches that have been developed such as affinity propagation are too expensive to be applied to datasets on the order of 10^6 ^points that are often generated by high throughput experiments.

**Results:**

To circumvent these limitations, we developed a new, unsupervised density contour clustering algorithm, called Misty Mountain, that is based on percolation theory and that efficiently analyzes large data sets. The approach can be envisioned as a progressive top-down removal of clouds covering a data histogram relief map to identify clusters by the appearance of statistically distinct peaks and ridges. This is a parallel clustering method that finds every cluster after analyzing only once the cross sections of the histogram. The overall run time for the composite steps of the algorithm increases linearly by the number of data points. The clustering of 10^6 ^data points in 2D data space takes place within about 15 seconds on a standard laptop PC. Comparison of the performance of this algorithm with other state of the art automated flow cytometry gating methods indicate that Misty Mountain provides substantial improvements in both run time and in the accuracy of cluster assignment.

**Conclusions:**

Misty Mountain is fast, unbiased for cluster shape, identifies stable clusters and is robust to noise. It provides a useful, general solution for multidimensional clustering problems. We demonstrate its suitability for automated gating of flow cytometry data.

## Background

Clustering is widely used for exploratory data analysis, with applications ranging from physics and biology to social sciences and psychology. In data intensive fields of biology, it is important to identify groups or clusters of data showing similar behavior. Many methods for clustering have been developed, which fall into two general categories: heuristic algorithms and model based analyses. In heuristic algorithms clustering is obtained either by optimizing a certain target function or iteratively agglomerating (or dividing) nodes to form bottom-up trees. Examples of these approaches include: K-means[1] and K-median [2] clustering, fuzzy K-means clustering [3], affinity propagation [4], spectral clustering [5,6], QT (quality threshold) clustering [7] and density contour clustering [8]. In contrast to heuristic methods, model-based clustering methods make inferences based on probabilistic assumptions about the data distribution. Gaussian or modified Gaussian mixture models [9] use the Expectation-Maximization algorithm [10-13] to find the parameters of the distributions that are fitted to the data. Then Bayesian information criterion (BIC) [14], Akaike information criterion (AIC) [13], integrated completed likelihood (ICL) [15] or other criterion is used to select the number of clusters.

Flow cytometry (FCM) is a commonly used technique to measure the levels of expression of multiple markers, such as specific proteins, in millions of cells. FCM data is typically analyzed by an attempt at visual selection of similar groups of data in 2 dimensional projections, a process referred to as gating. The visual identification of similar groups of data points, referred to in FCM as manual gating, is error-prone, non-reproducible, non-standardized, difficult to apply to more than two dimensions, and manpower-intensive, making it a limiting aspect of the technology [16]. Despite its widespread use, FCM lacks a fast and reliable method for automated analysis to parallel its high-throughput data-generation. The development of a reliable, heuristic clustering approach suitable for large datasets would significantly improve the value of FCM experiments and would have widespread application to other data-intensive biological clustering problems.

Automated FCM gating attempts using heuristic methods, such as K-means and fuzzy K-means [1,3,17-20] do not provide stable results. Different initial values for the algorithm, i.e. initial locations of the cluster centers, typically result in different clustering results. Often, with a poor set of initial values, the minimization of the target function falls into a local minimum and gives an undesirable clustering result. Furthermore, these methods work best with spherical or hyperspherical shaped clusters, a distribution often not observed in FCM datasets. Several other useful clustering algorithms based on pairwise comparisons, including linkage or Pearson coefficients method [21] and the affinity propagation method [4], are computationally too expensive to be used for FCM because the size of the pairwise distance matrix increases on the order of *n^2 ^*with the number of points. Classification and regression trees [22], artificial neural networks [23] and support vector machines [24,25] have also been used in the context of FCM analyses [26-29], but these supervised approaches require training data, which may not be available and may perform unreliably if the features of the experimental data diverge from the training set. Model-based approaches are slow, need user involvement and require assumptions about cluster distributions that limit their general utility [13,15]. A major problem of all practical approaches for unsupervised FCM cluster analysis remains the determination of the number of clusters. The use of BIC, AIC, ICL or other criterion can make the determination of cluster number unreliable (see Additional File 1).

To overcome these limitations of the above approaches, we have developed a new density contour clustering method that is particularly suitable for FCM data. In the early 1960's Boyell and Ruston[30], working on methods for storing topological data in a manner allowing efficient reconstruction, recognized that contour lines can be represented as a tree structure. This insight led to the idea of density contour clustering by finding the largest cross section of each histogram peak [8]. Jang and Hendry[31,32] used a density contour method for clustering galaxies, that in principle is most similar to our method. Their method is a modification of a method proposed by Cuveas et al.[33,34]. We have developed a new, fast density contour clustering method suitable for large, multi-dimensional datasets that will be compared with Jang and Hendry's method in Additional File 1. The method is unbiased for cluster shape and does not require global optimization of a multi-variable target function like other commonly used clustering methods do. The algorithm run time increases on the order of *n*. According to the tests on manually gated and simulated data the method provides correct clustering with correct number of clusters.

The Misty Mountain algorithm can be understood as the computational analogy of an airplane view of histogram terrain that is initially completely immersed in misty clouds. The mist is steadily removed from the top down by the sun, progressively uncovering clusters as peaks that pierce the mist. Eventually the merging points of two peaks, the highest saddle, is revealed. From there two peaks form one instead of two holes in the mist. As the level of the mist decreases, more and more summits and saddles are revealed and evaluated to determine the number of statistically distinct peaks and their extent.

## Results and Discussion

### Misty Mountain algorithm

The approach is briefly described here and more extensively in Methods. The multi-dimensional data is first processed to generate a histogram containing an optimal number of bins by using Knuth's data-based optimization criterion [35]. Then cross sections of the histogram are created. The algorithm finds the largest cross section of each statistically significant histogram peak. The data points belonging to these largest cross sections define the clusters of the data set.

**Figure 1:**
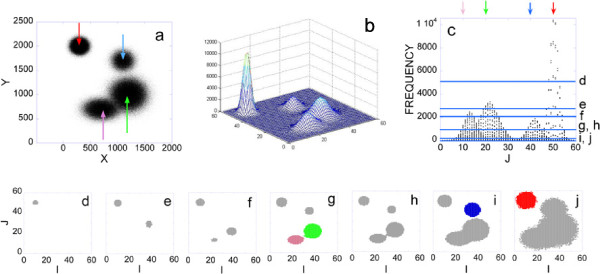
**Misty Mountain clustering of simulated FCM data**. a) Simulated 2 dimensional FCM data. Sum of four Gaussian distributions are simulated by using Monte Carlo techniques (see Methods). The center of each Gaussian is marked by a colored arrow. b) Two-dimensional histogram, *H(I,J) *of the simulated data is created by using an optimal 58 × 58 equally spaced mesh. c) Projection of the 2 dimensional histogram to the (J,FREQUENCY) plane. Blue lines: levels of histogram intersections shown in d)-j) subfigures. The frequencies at the intersections are: d) 5000, e) 2900, f) 2000, g) 757, h) 756, i) 103, j) 0.

To illustrate the method, we generated a simulated two-dimensional 10^6 ^FCM dataset with the respective histogram having four peaks (Figures 1a,b). Seven representative locations of the histogram intersection with a lowering plane are shown (Figures 1 c-j). Each cross section shows group(s) or aggregate(s) of those bins where the bin content is higher than the actual level of the cross section. With decreasing level, the number and size of the bin aggregates increase (Figures 1d-g). Then at the level of the highest saddle two bin aggregates coalesce (Figure 1h). At one level higher we have the largest, still separated bin aggregates (colored by pink and green in Figure 1g). The data points belonging to these two largest bin aggregates define the first two clusters of the cluster analysis. The blue colored aggregate in Figure 1i is shown just before coalescing with the gray colored aggregate. The data points belonging to this aggregate define the third cluster of the analysis. The cross section of the red peak is still separated and largest at *frequency = 0 *(colored by red in Figure 1j). The data belonging to the respective bin aggregate define the fourth cluster.

To realize the steps described above computationally, the algorithm uses a percolation theory based procedure[36,37] by labeling different bin aggregates of a histogram cross section by different integers. Then the algorithm comparatively analyzes pairs of consecutive cross sections to recognize coalescing bin aggregates. Assigning clusters to the coalescing bin aggregates requires the *L*_*p*1_-*L_s _*and *L*_*p*2_-*L_s _*relative heights of the two peaks that fuse both be statistically significantly greater than random fluctuations (see Methods). *L*_*p*1_, *L*_*p*2 _and *L_s _*are the heights of the fusing first and second peak and the saddle between them, respectively.

In the sample data, the algorithm assigned points to four clusters, requiring 14.7 seconds CPU time on a standard laptop PC. The characteristic properties of the assigned clusters such as cluster size and reliability of the assignation are shown in Table 1. These clusters contain 85% of all the 10^6 ^data points. Misty Mountain is a tight clustering method in that it does not force all points into clusters [38].

**Table 1:** Characteristics of the clusters assigned to data in Figure 1a

Color code	*L_p_*	*L_s_*	*C*	*f*
green	3385	756	313369	0.777

red	10706	0	300000	1

pink	2493	756	143539	0.697

blue	1911	102	94930	0.947

*L_p_*: height of the peak.

*L_s_*: height of the highest saddle next to the peak.

*C*: number of data points in the cluster.

*f*[= (*L_p_*-*L_s_*)/*L_p_*]: measure of separateness of the peak from nearby peak(s). The parameter estimates the reliability that an element of the cluster belongs to the respective population.

### Testing Misty Mountain algorithm

**Figure 2:**
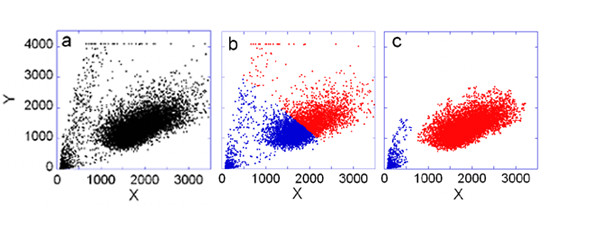
**Side scattering and forward scattering of U937 cells**. a) Experimental data. Side scattering is plotted against forward scattering. b) Result of cluster analysis by using the K-median clustering and spectral clustering with assuming 2 centers. c) Result of the cluster analysis by using the Misty Mountain method. Table 2 lists the characteristics of the resulting clusters. The data points assigned to the two clusters are marked by red and blue symbols.

We analyzed a flow cytometry dataset containing 9549 points representing the side scattering and forward scattering measurements obtained from U937 macrophage cells (Figure 2a). An expert in flow cytometry would interpret the large oval group as representing intact cells and would form a gate to separate these cells for further analysis from cellular debris. We first used K-median [2] and spectral clustering [5,6] algorithms. For K-median clustering we used simulated annealing [39] to find the global minimum of the target function, i.e. to find a stable solution of the clustering problem. Both of these conventional clustering methods gave similar erroneous results (Figure 2b). We next used the Misty Mountain algorithm to cluster these data. The respective optimal histogram contained 20 × 20 bins and there were 529 points in the most populated bin. Thus during the analysis, cross sections of the histogram were created at 529 levels. The elapsed CPU time of the cluster analysis was 0.28 sec. The result of the cluster analysis is shown in Figure 2c. These clusters contain 95.7% of all the data points, which are assigned at high confidence (Table 2).

**Table 2:** Characteristics of the clusters assigned to data in Figure 2a

Color code	*L_p_*	*L_s_*	*C*	*f*
red	430	5	8338	0.988

blue	529	5	804	0.991

(see legends to Table 1)

We next compared Misty Mountain with other state of the art flow cytometry automated gating algorthims using a variety of datasets (Table 3 and Additional File 1). The accuracy of these various algorithms was determined using expert manual gating to generate gold standards with 2 dimensional and 4 dimensional experimental datasets as well simulated 2 dimensional and 5 dimensional datasets having known cluster numbers. Algorithm run time was compared using these datasets as well as additional high dimensional experimental datasets for which a gold standard for accuracy was not generated. The accuracy of Misty Mountain was superior to that of all other methods tested. The speed of Misty Mountain was comparable to that of flowJo and orders of magnitude faster than other state of the art published methods. Extensive benchmarking suggests that Misty Mountain provides a significant improvement over the performance of other available methods.

**Table 3:** Summary of comparing Misty Mountain with state of the art flow cytometry specific clustering methods

Data set				Manually gated 2D barcoding^&^		Simulated 5D Gaussians	Simulated 2D non-convex		3D rituximab	4D GvHD	Manually gated 4D OP9	
Misty Mountain	accuracy	sens	(%)	100		100	100		-	-	100	
		
		spec	(%)	100		100	100		-	-	100	
	
	CPU	(sec)		10		196	6		0.3	0.8	3.6	

FLAME	accuracy	sens	(%)	20^a^	60^b^	-	0^d^*	100^d^	-	-	-	
		
		spec	(%)	33^a^	50^b^	-	0^d^*	100^d^	-	-	-	
	
		CPU	(sec)	5.10^4^		>3.10^5^	1.10^4^		10	360	1.4 · 10^4^	

flowClust	accuracy	sens	(%)	45^a^*	60^b^*	100^c^	0^c^*	100^d^	-	-	60^d^*	60*
		
		spec	(%)	60^a^*	55^b^*	100^c^	0^c^*	100^d^	-	-	75^d^*	38*
	
	CPU	(sec)		5.10^4^		4.10^4^	7200		43	480	3660	

flowMerge	accuracy	sens	(%)	25		100	0		-	-	80	
		
		spec	(%)	45		100	0		-	-	57	
	
	CPU	(sec)		1.3 · 10^5^		1.27 · 10^5^	7200		124	1020	8400	

flowJo	accuracy	sens	(%)	45		-	-		-	-	-	
		
		spec	(%)	47		-	-		-	-	-	
	
	CPU	(sec)		1-10		-	-		1-10	1-10	-	

^a ^optimal cluster number: 12

^b ^optimal cluster number: 24

^a^*optimal cluster number: 15

^b^*optimal cluster number: 22

^c ^optimal cluster number: 5

^c* ^optimal cluster number: 2

^d ^optimal cluster number: 1

^d* ^optimal cluster number: 4

* optimal cluster number: 8

^&^to save CPU time a data set, reduced by 80%, has been analyzed by FLAME, flowClust and flowJo

sens (sensitivity) = (# of correctly assigned clusters)/(# of clusters in gold standard)

spec(specificity) = (# of correctly assigned clusters)/(total # of assigned clusters)

Gold standards were independent expert manual clustering for experimental data and specified clusters for simulated data.

**Figure 3:**
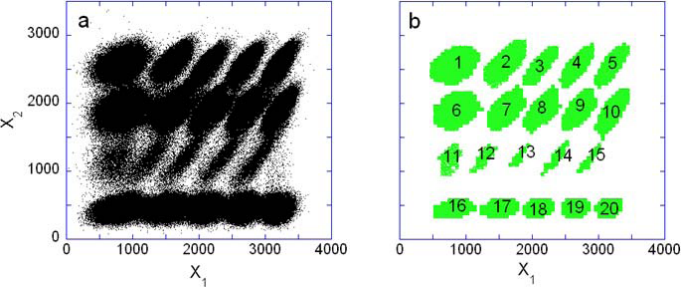
**Two-dimensional FCM data**. 853,674 U937 cells are stained by two florescence dyes, Pacific Blue and APC-Cy7-A. a) The fluorescence intensity of APC-Cy7-A is plotted against the fluorescence intensity of Pacific Blue. b) Result of the cluster analysis by using the Misty Mountain method. Each cluster is marked by a code number. Table in Additional File 2 lists the characteristics of the resulting clusters.

The performance of the Misty Mountain algorithm with a complex flow cytometry dataset consisting varying levels of two fluorophores, APC-Cy7-A and Pacific Blue-A, in 853,674 U937 cells is shown in Figure 3. The dataset in Figure 3a was generated for a barcoding experiment [40] in which different groups of cells were labeled with different concentrations of each fluorophore. The respective optimal histogram that was analyzed contained 52 × 52 bins. The most populated bin contained 4003 data points. Thus during the analysis, 4003 cross sections of the histogram were created. The elapsed CPU time of the cluster analysis was 9.8 sec. The results of the cluster analysis are shown in Figure 3b. The analysis identified 15 large clusters where the reliability of the cluster elements was from 0.75-0.98, and 5 small clusters with about 0.5 reliability. These clusters contained 87% of all the data points. The characteristic properties of the assigned clusters are listed in Table in Additional File 2. The last cluster in the table is a very small one and it is considered as noise (see Sec. Major and Small Peaks of the Histogram). In Additional Files 3 and 4 the analysis of an even more complex 3D barcoding experiment is shown.

As another example we analyzed one of the graft-versus-host disease (GvHD) data sets.

**Figure 4:**
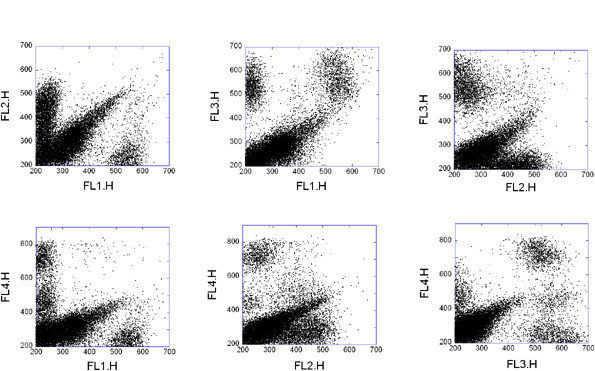
**Four-dimensional FCM data from the graft-versus-host-disease data set**. 10,463 peripheral blood mononuclear cells are stained by four florescence dyes: 1) CD4-FITC, 2) CD122-PE, 3) CD3-PerCP, 4) CD8-APC. At each axis of the plots the code number of the respective fluorescent stain is shown. Six 2D projections of the 4D data set are shown.

**Figure 5:**
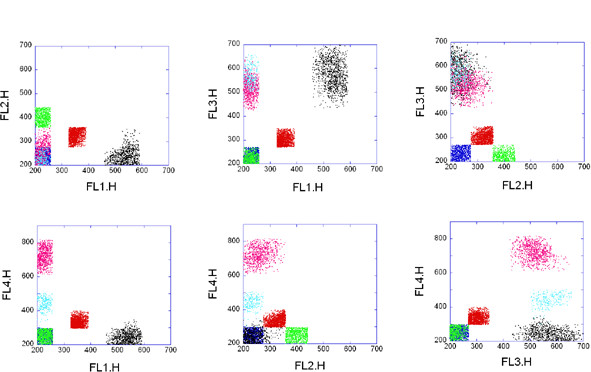
**Misty Mountain clustering of the graft-versus-host-disease data set**. 2D projections of the 4D clustering result are shown. Code numbers of clusters assigned by Misty Mountain algorithm: 1 (red); 2 (blue); 3 (green); 4 (black); 5 (rose); 6 (light blue). Table 4 lists the characteristics of the resulting clusters.

These 4D data sets have been made available [41] and used in a few flow cytometry analysis publications already [42]. The individual data files are available at: http://www.ficcs.org/data/data-files/. In our current example we used a data set from GVHD2.iso, Folder E#21 H06. Two dimensional projections of the data and the result of the clustering are shown in Figure 4 and 5, respectively. This data set is an example for overlapping populations. Misty Mountain algorithm assigned 6 clusters to the 4D GvHD data set within 0.8 sec. The analyzed histogram of the simulated data contained 8^4 ^bins. Since the populations are severely overlapping the assigned clusters contain only 29% of all the data points. Table 4 lists the characteristics of the clusters assigned by Misty Mountain. The low *f *values in Table 4 show that the histogram peaks belonging to cluster 1, 2 and 3 are seriously overlapping with nearby peak(s). In each of these cases Misty Mountain assigns cluster to a histogram cross section that is close to the top of the respective peak and thus the number of histogram bins assigned to these seriously overlapping clusters is low. The above two data sets are also analyzed by state of the art clustering methods in Additional File 1 and compared with the results of Misty Mountain clustering.

**Table 4:** Characteristics of clusters assigned by Misty Mountain to the 4D GvHD data in Figure 4.

Code #	*L_p_*	*L_s_*	*C*	Bin #	*f*
1	1541	1033	1542	1	0.33

2	1115	1033	1116	1	0.074

4	230	25	1011	11	0.891

3	889	804	890	1	0.096

5	175	30	858	8	0.829

6	132	30	265	3	0.773

(see legends to Table 1)

Bin #: number of histogram bins containing the points of a cluster

We also compared the performance of the various gating algorithms using a dataset from 4D bone-marrow derived mouse stromal cells (OP9 cells) stained with antibodies for CD45, Gr1, Mac1 and CD19. Two experts manually gated this experiment obtaining identical results. Misty Mountain gave results identical to that of the experts, unlike the other automated gating methods (Table 3 and in Additional File 1, Figures AF14-18, Table AF13-18). In order to test algorithm performance we used a variety of other experimental and simulated data sets with biologically interesting populations such as low density, overlapping and non-convex populations. Comparisons were made using simulated 2 dimensional and 5 dimensional data and additional experiments with 3 dimensional and 4 dimensional data (Additional File 1). These results all strongly support the improved accuracy and utility of the Misty Mountain algorithm relative to other state of the art methods.

Studies were done to evaluate the time complexity of the Misty Mountain algorithm. These simulations revealed that at fixed bin number the overall run time for the composite steps of the algorithm increases linearly by the number of data points. Also an increase in the run time was detected with increasing dimensionality of the data space (see Additional File 5). The number of clusters did not alter the computation time (Additional File 5).

The Misty Mountain algorithm can be applied to analyze other than FCM data when the data set is large enough to construct an adequate histogram. For example in astrophysics it can be used for unsupervised recognition of star/galaxy clusters, or in social sciences to analyze questionnaires and identify groups with common interests/opinions.

### Implementation

The implementation, instruction and the input data files of all the examples analyzed in this study are available in Additional Files 6, 7 and 8.

### PCA- Misty Mountain algorithm for high dimensional data

The current version of the Misty Mountain algorithm software uses direct analysis for data having up to 5 dimensions. Some flow cytometry datasets may have up to twelve or even more dimensions. One can set the critical dimension higher than 5, however the run time, the number of data points needed for an adequate histogram and the memory requirement for storing the histogram increases super linearly with increasing dimension. As another option, we have combined the Misty Mountain algorithm with principal component analysis (PCA) [43]. In order to analyze higher than 5 dimensional data, we use PCA to project the high dimensional data into a 5 dimensional subspace. The subspace is spanned by 5 eigenvectors belonging to the 5 largest eigenvalues of the covariance matrix of the data. Then Misty Mountain analysis is performed on the projected data. Finally the points of the assigned clusters are back-projected into their original position in the data space. This procedure is demonstrated on a simulated 10 dimensional data set containing points that distributed as the sum of 8 distorted-Gaussians. The parameters of the distorted-Gaussians (mean and standard deviation of the distributions) are listed in the table in Additional File 9. By using PCA, the simulated data are projected into the 5D subspace where Misty Mountain clustering is performed. The points of the assigned 8 clusters are back-projected to their original position in the 10D data space. Table in Additional File 10 lists the center coordinates of the assigned clusters. As a demonstration of correct clustering these cluster centers are very close to the means of the respective distorted-Gaussians. It is important to note that the projection of the data into the 5D subspace may bring some of the otherwise separated histogram peaks so close to each other that the number of clusters assigned by the Misty Mountain algorithm becomes less than the true value. This happens with higher frequency when the data histogram contains many, broad peaks. Finally it is important to note that the optimal choice for the critical dimension depends on the actual number of the data points, i.e. one should be able to create an adequate histogram from the data at the critical dimension.

### Advantages and limitations of Misty Mountain algorithm

Advantages:

1) Misty Mountain algorithm is unbiased for cluster shape.

2) it is robust to noise,

3) it is fast,

4) it is unsupervised. It does not need estimation for cluster number.

5) the computation time linearly increases with the number of data points

Limitations:

1) Misty Mountain algorithm identifies two closely situated populations as one when the respective histogram has only one peak

2) it identifies two populations as one when *L_p_*-*L_s _*is comparable with the standard deviation of *L_p_*-*L_s_*. (*L_p _*is the bin content at the smaller histogram peak, and *L_s _*is the bin content at the saddle between the two histogram peaks.)

3) the computation time, the number of data points needed for an adequate histogram and the memory requirement for the histogram super linearly increase with the dimension of the data space

Misty Mountain provides a useful, general solution for multidimensional clustering problems. It can be easily adapted to address diverse large dataset clustering problems in computational biology. It is particularly suitable for automated gating of FCM and should improve the ability to interpret experimental data in this field.

## Conclusions

In biology, measurements on a single object (such as a cell or image) are frequently represented by a point in a multi-dimensional space where the coordinates of the point refer to the measured values. With the advent of high-throughput assays, these experiments can generate datasets comprising millions of points. Clusters of points may be thought of as regions of high density separated from other such regions of low density. We describe a fast algorithm that automatically identifies clusters of data points showing similar values. The three major steps of the algorithm are: i) The multi-dimensional data is first processed to generate a histogram containing an optimal number of bins. ii) The cross sections of the histogram are created. iii) The algorithm finds the largest cross section of each statistically significant histogram peak. The data points belonging to these largest cross sections define the clusters of our data set.

While the idea of clustering by using a density histogram is old, the present implementation results in particularly fast clustering that is useful for data-intensive computational biology applications. Misty Mountain clusters 10^6 ^data points in 2D data space in about 15 seconds on a standard laptop PC. The run time linearly increases with the number of data points. Unlike other commonly used clustering methods, Misty Mountain is not model-based, unsupervised and does not require global optimization of a multi-variable target function. Without making strong assumptions, this method provides fast and accurate clustering. The algorithm is general, but was motivated by the need for an unbiased automated method for analysis of flow cytometry (FCM) data.

## Methods

In the previous sections we gave a qualitative description of the Misty Mountain algorithm. In order to help to understand the logic of the algorithm, we discuss its key features in detail.

**Figure 6:**
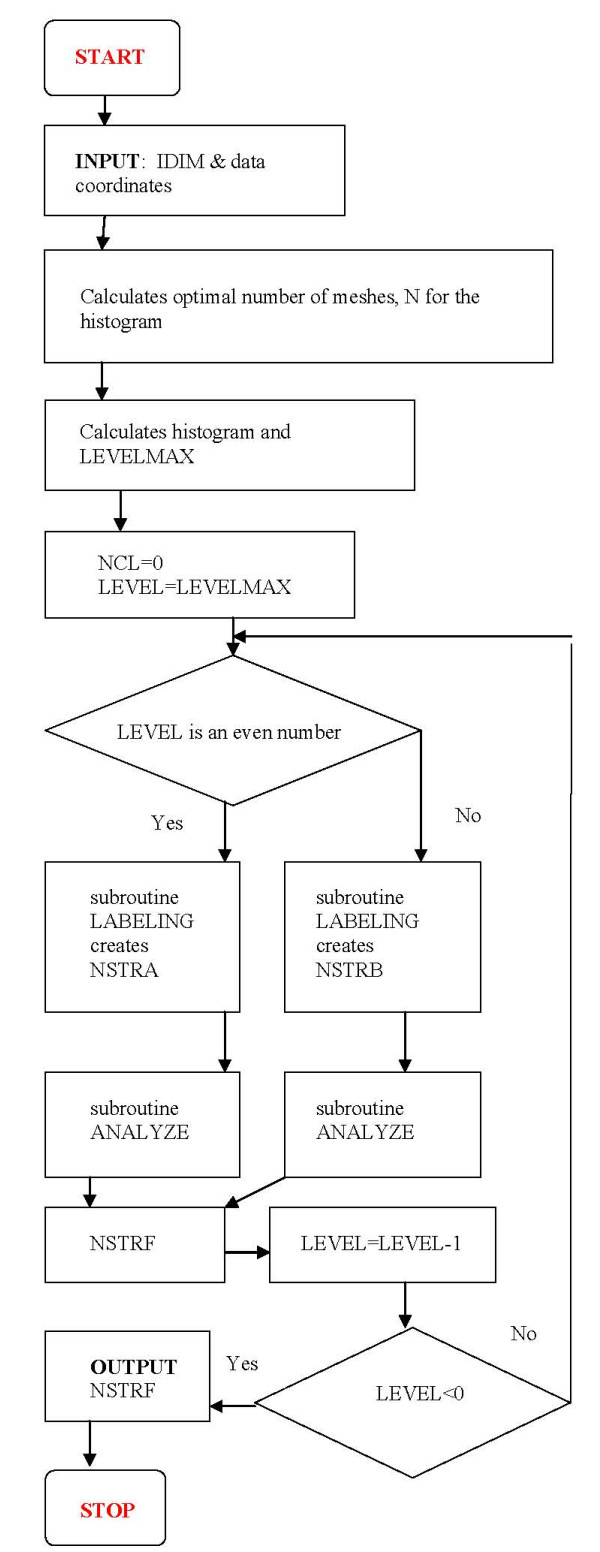
**Flow chart of the main part of the Misty Mountain program**. IDIM - dimension of the data space. N - the number of equidistant meshes along each coordinate for creating optimal histogram. LEVELMAX - highest frequency of the histogram. LEVEL - frequency where the actual cross section is created. NCL - actual number of (major and small) histogram peaks. NSTRA and NSTRB - matrices of labeled aggregates of two consecutive cross sections. NSTRF - structure matrix that stores the largest separate aggregates belonging to the major peaks (see e.g. colored aggregates in Figure 1d-j).

The main part of the program reads in the coordinates of the data points, creates an optimal histogram from the data, analyses the consecutive cross sections of the histogram by calling two major routines - LABELING and ANALYZE -, and finally outputs the result (see flowchart in Figure 6). These major steps of the program are discussed below.

### Histogram Optimization

By using the Bayesian framework Knuth [35] proposed an optimal data-based binning for histograms. He derived the posterior probability, *p *for the number of bins of similar shape at given data, *d*. If there is similar number of bins, *N *along each coordinate axis the logarithm of the posterior probability is:

$$\begin{array}{l} \log p(N|\underline{d})=\\ n\log N^{D}+\log\Gamma \left(0.5N^{D}\right)-N^{D}\log\Gamma (0.5)-\log\Gamma (n+0.5N^{D})+\sum_{k=1}^{N^{D}}\log\Gamma (n_{k}+0.5)+const. \end{array}$$

where *n *is the number of data, *n_k _*is the number of data in the *k*^th ^bin, and *D *is the dimension of the data space. The *N *that maximizes this probability is the optimal bin number along each coordinate axis. There are other optimal data based binning methods such as Wand's method [44]. We prefer using Knuth's method because its implementation is particularly easy for any dimension of data.

### The LABELING routine

The LABELING routine separately analyzes each cross section of the histogram. As an example let us consider a two dimensional histogram (e.g. Figure 1b). Each cross section of the histogram is mathematically represented by an *NxN *square matrix where the *I,J*-th element of the matrix is equal 1 if the respective bin content is higher than *LEVEL *(the frequency at the actual cross section) otherwise it is 0. In this matrix the cross section of a peak appears as a group or aggregate of 1's. The aim of the aggregate labeling algorithm is to assign the same positive integer to the same aggregate. On the other hand different aggregates will be labeled by different integers. The *NxN *label matrix, *NSTRA *is created in three steps.

Step 1. Initialization of the label matrix.

*NSTRA(I,J) = NBIN *if the content of the I,J-th bin is larger than the level of the cross section, otherwise *NSTRA(I,J) = 0*. *NBIN = NxN *is the number of bins. Set the aggregate counter zero, i.e.: *IL = 0*.

Step 2. First scanning of the label matrix.

Starting from the first matrix element, *NSTRA*(1,1) let us scan the matrix from left to right and from the top to the bottom. Let us change the values of the matrix elements according to the following rules:

a) If *NSTRA*(*I*, *J*) = 0 then it remains zero

b) If *NSTRA*(*I*, *J*) = *NBIN*, and *NSTRA*(*I*-1, *J*-1), *NSTRA*(*I*-1, *J*), *NSTRA*(*I*-1, *J+*1) and *NSTRA*(*I*, *J*-1) matrix elements (if they exist) are equal to 0, then first let us increase the value of *IL *by 1; second change the value of *NSTRA*(*I*, *J*) from NBIN to *NSTRA*(*I*, *J*) = *IL*; and, finally, let the *IL*-th element of the *ICOUNT *vector equal to *IL*.

c) If *NSTRA*(*I*, *J*) = *NBIN*, and any of the *NSTRA*(*I*-1, *J*-1), *NSTRA*(*I*-1, *J*), *NSTRA*(*I*-1, *J+*1) and *NSTRA*(*I*, *J*-1) matrix elements (if they exist) are not equal to 0 then we determine the proper aggregate label for these non-zero neighbor matrix elements by applying routine *CLASSIFY *(described in *Step 3*). Then we select the smallest of the proper labels, called *JM*, and we set

$$\begin{array}{l} ICOUNT(NSTRA(I,J))=JM\\ ICOUNT(NSTRA(I-1,J-1))=JM\\ ICOUNT(NSTRA(I-1,J))=JM\\ ICOUNT(NSTRA(I-1,J+1))=JM\\ ICOUNT(NSTRA(I,J-1))=JM\\ NSTRA(I,J)=JM \end{array}$$

Step 3: Second scanning of the label matrix.

After the first scanning, an aggregate may have more than one label. During the second scan, we assign a single label to each element of an aggregate. In this scan the zero elements remain unchanged, while the new value of the nonzero element *NSTRA*(*I*, *J*) is determined by means of the following procedure called *CLASSIFY*:

$$\begin{array}{cc} 1 & \begin{array}{l} LIJ=NSTRA(I,J)\\ MS=LIJ\\ LIJ=ICOUNT(MS)\\ IF(MS.NE.LIJ)GOTO1\\ NSTRA(I,J)=LIJ \end{array} \end{array}$$

This simple procedure finds the smallest label among the labels of the aggregate where the *I,J*-th bin is situated. The labeling routine is similar to the one used in percolation theory [36,37] for labeling spin clusters. The difference is that in Step 2b and 2c in spin cluster labeling, usually only two nearest neighbors: *NSTRA(I-1,J) *and *NSTRA(I,J-1)*, of the *I,J*-th matrix element are considered. In our algorithm, we also consider two next nearest neighbor matrix elements, *NSTRA(I-1,J-1) *and *NSTRA(I-1,J+1)*. By using this important modification elongated slanted aggregates are properly labeled. The FORTAN source code of the LABELING routine is able to label bin aggregates of any dimension.

### The ANALYZE routine

**Figure 7:**
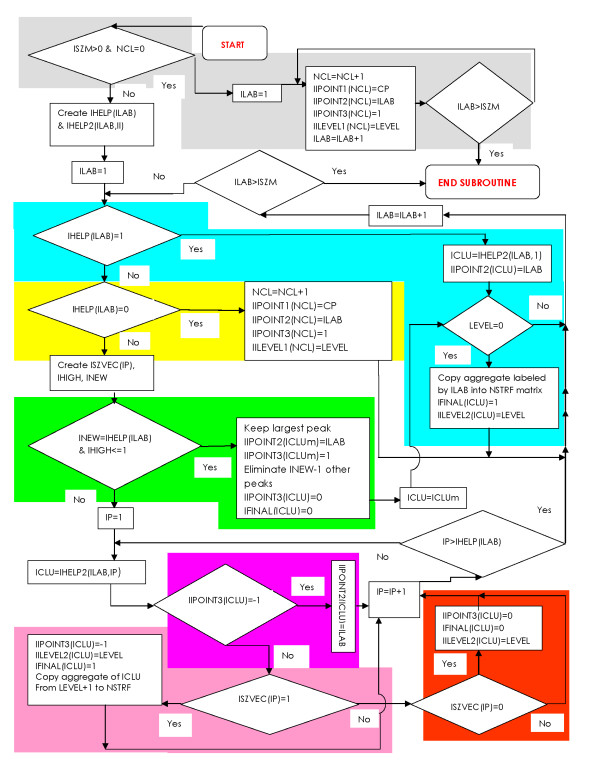
**Flow chart of the ANALYZE routine of the Misty Mountain program**. ISZM - the largest label of the aggregates in a cross section. (For simplicity this flowchart assumes that ISZM is also the number of aggregates. In reality ISZM is frequently larger than the number of aggregates.) ICLU - code number of a peak. ILAB - label of an aggregate. IP - counter of peaks belonging to the same aggregate. CP - characteristic position of a peak. IIPOINT1(ICLU) - characteristic position of the ICLU-th peak. IIPOINT2(ICLU) - label of the aggregate at the ICLU-th peak. IIPOINT3(ICLU) = T - the type of the ICLU-th peak: *T *= 1 - single peak, *T *= 0- merged small peak, *T *= -1 - merged major peak. The values of the IIPOINT2 and IIPOINT3 vector elements are updated at each level. IILEVEL1(ICLU) - level at the top of the ICLU-th peak. IILEVEL2(ICLU) - level of the saddle where the single ICLU-th peak coalesces with another peak. IHELP(ILAB) - number of characteristic peak positions falling into an aggregate labeled by ILAB. IHELP(ILAB,IP) - the code number of the peak belonging to the IP-th characteristic peak position in the aggregate labeled by ILAB. IFINAL(ICLU) = 0 - when ICLU-th peak is eliminated from the analysis. IFINAL(ICLU) = 1 - when the aggregate belonging to the ICLU-th peak is copied into NSTRF. ISZVEC(IP) = -1 - the IP-th peak has merged with other peak at a higher level. ISZVEC(IP) = 0- the IP-th peak was a small single peak at the previous level. ISZVEC(IP) = 1 - the IP-th peak was a major single peak at the previous level. INEW - number of single peaks merging with each other at the current level. IHIGH - number of major peaks from the INEW single peaks. Other notations are at the legends to Figure 6.

This routine performs a comparative analysis of the actual and previous cross sections, and stores the largest but still separated aggregates of the major peaks. It also recognizes and eliminates small noisy peaks from the analysis. The distinction between small and major peaks is explained below in Sec. Major and Small Peaks of the Histogram. The flowchart in Figure 7 shows the logic of the ANALYZE routine.

First we give a brief description of the flowchart in Figure 7. The cross sections of the histogram are created consecutively from the highest to the lowest level, i.e. from LEVELMAX to 0. In the grey region of the flow chart aggregates emerging at LEVELMAX are handled. When a new aggregate appears at a lower level the yellow part of the flow chart is active. The cyan colored part of the flow chart is active when a single peak that emerged at a previous level belongs to the aggregate. The rest of the flow chart is active when more than one peak belongs to the aggregate. The green colored part is active when at the previous level every peak was single and no more than one of them was major peak. The red, purple and pink colored parts are active when at the previous level either not every peak was single or more than one single peak were major.

Now in the rest of this section a more detailed description of the flowchart (Figure 7) is given. At the highest level, one or more aggregates appear in the cross section. The counter of the peaks NCL is increased from zero, and the types and positions of the emerging peaks are registered in three vectors:

IIPOINT1(ICLU) - location (or characteristic position) of the emerging ICLU-th peak

IIPOINT2(ICLU) - label of the aggregate of the ICLU-th peak

IIPOINT3(ICLU) = 1 - type code of a single peak (the other two type codes are defined below). In the flow chart (in Figure 7) these steps are highlighted by grey.

At every other cross section level the analysis of the labeled aggregates starts by creating the IHELP vector. IHELP(ILAB) is the number of characteristic peak positions that fall into the aggregate labeled by ILAB. There are three possibilities:

a) There is no characteristic peak position in the aggregate labeled by ILAB.

This signifies that a peak is just emerging. In this case NCL is increased by one and proper values are assigned to the NCL-th elements of the IIPOINT1, IIPOINT2 and IIPOINT3 vectors. In Figure 7 the respective part of the flow chart is highlighted by yellow.

b) There is one characteristic peak position in the aggregate labeled by ILAB.

This means that the respective peak, with code number ICLU, emerged in one of the previous cross sections, and it is still a single peak. Thus the peak type IIPOINT3(ICLU) remains equal to 1. If the peak remains single until the lowest level of the cross section (i.e. until LEVEL = 0) the aggregate belonging to this peak is copied into the final label matrix, NSTRF where it is labeled by ICLU. In the NSTRF matrix we store the final result of our cluster analysis. In the flow chart (in Figure 7) the above described steps are highlighted by cyan.

c) There are more than one characteristic peak positions in the aggregate labeled by ILAB.

This is the most important part of the algorithm that handles the merger of major peaks and the elimination of small noisy peaks. The counter of the peak positions falling into the aggregate is denoted by IP.

The analysis of the aggregate starts by creating the ISZVEC vector. The IP-th element of the vector refers to the type of the respective peak at the previous cross section: ISZVEC(IP) = -1 when the IP-th peak has merged with other peak(s), ISZVEC(IP) = 0 when the IP-th peak was a small single peak, and ISZVEC(IP) = 1 when the peak was a single major peak. The number of 1's in ISZVEC is denoted by IHIGH, while the number of 0's and 1's is denoted by INEW.

c1) If at the previous level every peak was single - *INEW *= *IHELP*(*ILAB*), and no more than one of them was major peak - *IHIGH *≤ 1.

In this case the highest peak, encoded by ICLUm, is retained, while all the other peaks are eliminated, i.e.: IIPOINT3(ICLUm) = 1 and IIPOINT3(ICLU) = 0 for all the remaining small peaks. In Figure 7 the respective part of the flow chart is highlighted by green.

This strategy is particularly useful at cross sections where a major peak appears. Frequently, as a first sign of a major peak, small nearby aggregates appear that merge at lower levels. This is also our usual strategy for retaining major peaks, while eliminating the frequently appearing small noisy peaks.

If LEVEL = 0 the aggregate belonging to the retained ICLUm-th peak is also copied into the final label matrix, NSTRF (see part of the flow chart highlighted by cyan).

c2) In all other cases - either not every peak was single at the previous level or more than one single peak was major - there are three options.

c21) Small and previously single peaks are eliminated, i.e. the value of the respective elements of IIPOINT3 vector change from 1 to 0. This part of the flow chart is highlighted by red.

c22) Major and previously single peaks become merged peaks, i.e. the value of the respective elements of IIPOINT3 vector change from 1 to -1. The aggregates belonging to these peaks at the previous cross section are copied into the final label matrix, NSTRF. The respective part of the flow chart is highlighted by pink.

c23) Handling of previously merged peaks is shown and highlighted by purple in the flow chart.

### Major and Small Peaks of the Histogram

A histogram contains major peaks such as the four peaks in Figure 1b and small peaks that are superimposed on the major peaks. A small peak is the consequence of the fluctuation of the number of data points in the respective bins. One can observe this fluctuation of bin contents by comparing the histograms of repeated experiments.

The fluctuation of the bin content can be estimated as follows.

First we point out that the content of each bin follows binomial distribution. Let us assume that we measure *n *cells to create our FCM data set. The probability that the measured fluorescent intensities of a cell falls into the *ε*-th bin is *p_ε_*. If the measurements on different cells are statistically independent events the probability that out of *n *measurements the result of *b *measurements will fall into the *ε*-th bin and *n-b *measurements will fall out of the *ε*-th bin is: $P(b,n|p_{\varepsilon })=\left(\begin{array}{c} n\\ b \end{array}\right)p_{\varepsilon }^{b}{\left(1-p_{\varepsilon }\right)}^{n-b}$

This is the binomial distribution. If the mean bin content, <*b*> is larger than 10 the binomial distribution can be approximated by its limit: the Poisson distribution [45]. The mean of the Poisson distribution can be estimated by the average of the contents in the actual and nearest-neighbor bins $\bar{b}$, while the standard deviation of the Poisson distribution by the square root of this average $\sqrt{\bar{b}}$.

Every time when two or more aggregates merge we have to decide if the merging aggregates belong to small and/or major peaks. A peak is considered major if: $L_{p}-L_{s}>2\sqrt{{\bar{b}}_{p}+{\bar{b}}_{s}}$ where *L_p _*and *L_s _*are the peak height and the height of the saddle between the merging peaks, respectively. On the other hand a peak is considered small if $0<L_{p}-L_{s}\leq 2\sqrt{{\bar{b}}_{p}+{\bar{b}}_{s}}$. When ${\bar{b}}_{p}<10$ the Poisson approximation fails and the respective peak is always considered small.

### Simulation of Data

In each simulated data set the data points follow a sum of regular or distorted-Gaussian distributions. As the first step of the simulation the means and standard deviations of the distorted-Gaussians are arbitrarily or randomly assigned along each coordinate axis. Also two coordinate axes are randomly selected to each distorted-Gaussian; directions along which the Gaussian will be distorted. *X_IK_*, the K-th coordinate of a data point belonging to the I-th distorted-Gaussian is simulated as follows:

$$\begin{array}{l} X_{IK}=X_{IK}^{mean}+\Delta \cdot SD_{IK}\text{ if }K\neq K_{1}^{I}\text{ and }K\neq K_{2}^{I}\\ X_{IK}=X_{IK}^{mean}+\Delta_{1}\cdot SD_{IK}\text{ if }K=K_{1}^{I}\\ X_{IK}=X_{IK}^{mean}+\Delta \cdot SD_{IK}+s\cdot {\left[\Delta_{1}\cdot SD_{IK_{1}^{I}}\right]}^{2}\text{if }K=K_{2}^{I} \end{array}$$

where Δ and Δ_1 _is a normal deviates generated by the Box-Muller method [46], $X_{IK}^{mean}$ is the K-th coordinate of the mean of the I-th distorted-Gaussian and *SD_IK _*is the standard deviation of the I-th distorted-Gaussian along the K-th axis, while $K_{1}^{I}$ and $K_{2}^{I}$ are the first and second axes, respectively that randomly selected to the I-th distorted-Gaussian. Parameter *s *scales the strength of the distortion. In the case of our 2D and 10D simulations *s *= 0.002 and 0.004 have been used. Note that by using the above procedure one can simulate the sum of regular Gaussian distributions by setting the distortion parameter *s = 0*.

## Authors' contributions

IPS implemented the algorithm and performed all analyses. Both authors collaborated on the algorithm design and the manuscript. Both authors read and approved the final manuscript.

## Supplementary Material

Additional file 1 **Comparing Misty Mountain clustering with other state of the art clustering methods**. Click here for file

Additional file 2 **Table of cluster characteristics assigned to data in **Figure 3a. (see legends to Table 1 - main text) The i^th ^coordinate of the center of each cluster was calculated by averaging the i^th ^coordinates of the *C *cluster elements: $X_{i}^{center}=\sum_{j=1}^{C}X_{i}(j)/C$ Click here for file

Additional file 3 **Figure of three-dimensional FCM data**. 853,674 U937 cells are stained by three florescence dyes, Pacific Blue, ALEXA-350-A and APC-Cy7-A. The fluorescence intensities of these dyes are plotted on the *X*_1_, *X*_2 _and *X*_3 _axes, respectively. By creating equidistant meshes of the *X*_3 _axis from the lowest to the highest intensity the three dimensional data space is divided into 46 slices. Left panels refer to the a) 6^th^, b) 16^th^, c) 26^th ^and d) 35^th ^slice of the data space. Right panels show the respective slices from the result of the cluster analysis. In the four slices all the assigned 89 clusters are represented. Each cluster is colored by red and marked by a code number. Code number 1 refers to the cluster containing the largest number of data points, number 2 to the second largest, etc. Virtually disconnected clusters with similar code number are in reality connected at a nearby slice. Table in Additional File 4 lists the characteristics of the resulting clusters. The optimal histogram contained 46 × 46 × 46 bins, and the cluster analysis required 11.2 seconds CPU time. Click here for file

Additional file 4 **Table of cluster characteristics assigned to data in **Additional File 3. (see legends to Table in Additional File 2) Click here for file

Additional file 5 **Figures of simulation results on Misty Mountain clustering time complexity**. Misty Mountain clustering has been performed on a series of simulated datasets. a) The run time of each analysis (dot) is plotted against the number of respective data points. Red dots: the datasets simulate the same 4 Gaussians in 2D (as in Figure 1a) but contain different number of points. Green dots: the datasets simulate the same 7 Gaussians in 4D but contain different number of points. Blue dots: the datasets simulate the same 5 Gaussians in 5D but contain different number of points. At a fixed bin number the run time increases linearly with the number of data points. b) The run time is plotted against the number of simulated Gaussians. In each of these simulations the number of data points were kept the same: 100,000, while the dimension of the data space was: 2D (red curve), 3D (blue curve), 5D (green curve). Click here for file

Additional file 6 **Implementation, instruction and data files**. Click here for file

Additional file 7 **Implementation, instruction and data files**. Click here for file

Additional file 8 **Implementation, instruction and data files**. Click here for file

Additional file 9 **Table of the parameters of 8 distorted-Gaussian distributions simulated in 10D**. The sum of 8 distorted-Gaussian distributions was simulated in 10D space with distortion parameter s = 0.004 (see Methods). The center coordinates, $X_{i}^{mean}$ and the standard deviations, *SD_i _*of each distorted-Gaussian were randomly generated within (0,1000) and (0,200) intervals, respectively (see Methods). Click here for file

Additional file 10 **Table of center coordinates of clusters assigned within the 5D subspace of the 10D simulated data**. The Misty Mountain algorithm assigned 8 clusters to the 10D simulated data when the data were projected into a 5D subspace. The analyzed 5D histogram of the projected data contained 8^5 ^bins. The cluster elements were back-projected into the 10D data space. The clusters contain 72.7% of all the data points. The computation time was 9.4 sec. The Table lists the coordinates of each cluster center. The i^th ^coordinate of the center of each cluster was calculated by averaging the i^th ^coordinates of the *C *cluster elements: $X_{i}^{center}=\sum_{j=1}^{C}X_{i}(j)/C$. 8 out of the 8 cluster centers coincide with the centers of the 8 simulated distorted-Gaussians (listed in Table in Additional File 9). Click here for file
