# Supplementary material for: Misty Mountain clustering: application to fast unsupervised flow cytometry gating
Source: BMC Bioinformatics. 2010 Oct 9;11:502. doi: 10.1186/1471-2105-11-502 (PMC2967560; doi:10.1186/1471-2105-11-502)
Supplement: Additional file 1 — Comparing Misty Mountain clustering with other state of the art clustering methods. [file 1471-2105-11-502-S1.DOC]

Additional File 1

Comparing Misty Mountain clustering with other state of the art clustering methods

Table of contents

1. Comparison with other density contour clustering method

2. Comparison with other methods optimized for clustering FCM data.

2.1 Clustering 2D barcoding data by different methods

2.1.1 Manual clustering

2.1.2 Misty Mountain clustering

2.1.3 Clustering by FLAME mixture model

2.1.4 Clustering by flowClust mixture model

2.1.5 Clustering by flowMerge mixture model

2.1.6 Clustering by flowJo flow cytometer’s clustering program

2.1.7 Clustering 2D barcoding data. Concluding remarks

2.2 Clustering 3D rituximab data by different methods

2.2.1 Rituximab data

2.2.2 Misty Mountain clustering

2.2.3 Clustering by FLAME mixture model

2.2.4 Clustering by flowClust mixture model

2.2.5 Clustering by flowMerge mixture model

2.2.6 Clustering 3D rituximab data. Concluding remarks

2.3 Clustering 4D GvHD data by different methods

2.3.1 GvHD data

2.3.2 Misty Mountain clustering

2.3.3 Clustering by FLAME mixture model

2.3.4 Clustering by flowClust mixture model

2.3.5 Clustering by flowMerge mixture model

2.3.6 Clustering by flowJo flow cytometer’s clustering program

2.3.7 Clustering 4D GvHD data. Concluding remarks

2.4 Clustering 4D OP9 data by different methods

2.4.1 OP9 data

2.4.2 Manual clustering

2.4.3 Misty Mountain clustering

2.4.4 Clustering by FLAME mixture model

2.4.5 Clustering by flowClust mixture model

2.4.6 Clustering by flowMerge mixture model

2.4.7 Clustering 4D OP9 data. Concluding remarks

2.5 Clustering 5D simulated data by different methods

2.5.1 Simulation of data

2.5.2 Misty Mountain clustering

2.5.3 Clustering by FLAME mixture model

2.5.4 Clustering by flowClust mixture model

2.5.5 Clustering by flowMerge mixture model

2.6 Clustering 2D simulated data. Case of non-convex shape cluster

2.6.1 Simulation of data

2.6.2 Misty Mountain clustering

2.6.3 Clustering by FLAME mixture model

2.6.4 Clustering by flowClust mixture model

2.6.5 Clustering by flowMerge mixture model

2.7 Comparison of clustering methods. Concluding remarks

1. Comparison with other density contour clustering method

Jang and Hendry [1, 2] used a density contour method for clustering galaxies, that in principle most similar to our method. Their software is not publicly available and not optimized for the analysis of FCM data. We can give, however, a general comparison between their clustering method and Misty Mountain. The implementation is based on the ideas described by Cuveas et al.[3, 4]. Jang and Hendry calculate the histogram by using a fast Fourier transform method of Silverman[5] that requires $O(\prod_{i=1}^{d} m_{i}\log m_{i})$to compute. On the other hand we use Knuth’s method[6] that requires only $O(\prod_{i=1}^{d} m_{i})$ to compute. Here *d* is the dimension of the data space and $m_{i}$ is the number of bins along the *i*^th^ axis.

During the analysis of a cross section of the histogram Jang and Hendry use their own method that requires $O(k_{m}\log k_{m})$to compute, where $k_{m}$ is the total number of histogram bins. However we use for the analysis of the cross section Hoshen and Kopelman’s method[7] that requires $O(3^{d}\cdot k_{m})$ to compute.

Because of these differences analyzing a data set of 10^5^ points our program is 2-3 orders of magnitude faster than Jang and Hendry’s (personal communication). We should mention another important difference between the two methods. Jang and Hendry arbitrarily selects the total number of histogram bins, $k_{m}$, while in our case it is the result of a data based optimization proposed by Knuth[6].

2. Comparison with other methods optimized for clustering FCM data.

Experimental and simulated flow cytometry data sets are analyzed in this Section by using the following publicly available clustering methods: flowClust (http://www.bioconductor.org/packages/2.2/bioc/html/flowClust.html), FLAME (<http://www.broadinstitute.org/cancer/software/genepattern/modules/FLAME/>), flowMerge (<http://www.bioconductor.org/packages/devel/bioc/html/flowMerge.html>) and the clustering software provided by flowJo (http://www.flowjo.com/v8/html/cluster.html) Except for flowMerge we use the default parameter settings of these programs. The same data sets are also analyzed by Misty Mountain clustering. Manually gated or simulated data sets serve as gold standards of correct clustering and we compare the clustering results to these standards. When the analyzed data set is not a standard one we compare the clustering results to each other. The analyzed data sets can be found in Additional Files 6, 7 and 8.

FLAME and flowClust are model based clustering algorithms that were recently developed to automate FCM data analysis[8, 9]. FlowClust is based on multivariate t mixture model with the Box-Cox transformation. This approach generalizes Gaussian mixture models by modeling outliers using t distributions and allowing for clusters taking non-ellipsoidal convex shapes upon proper data transformation. FLAME on the other hand presents a direct multivariate finite mixture modeling approach, using also skew and heavy-tailed distributions, without the need for projection or transformation. In both of these clustering methods parameter estimation is carried out using an Expectation-Maximization (EM) algorithm.

FlowMerge is based on the flowClust_BIC_ solution, thus retaining the property of good fit to the distribution, while simultaneously eliminating ambiguity associated with multiple overlapping components representing the same cell subpopulation.

FlowJo’s clustering algorithm has been developed by Dr. Mario Roederer and it is based on his Probability Binning Algorithm [10-12] developed for the statistical comparison of samples of FCM data.

**2.1 Clustering 2D barcoding data by different methods**

***2.1.1 Manual clustering***

The 2D barcoding data shown in Figure 3a (main text) has been analyzed by an expert uninvolved in this study and blinded to the computational results On the density plot representation of the 2D barcoding data our expert gated 20 clusters within 6 minutes (Figure AF1a).


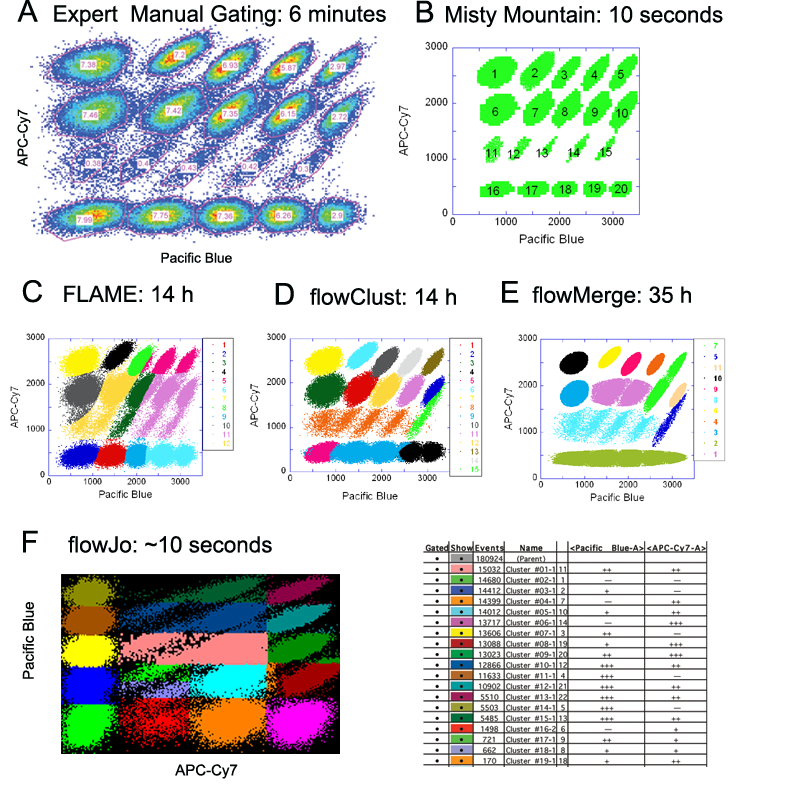


**Figure AF1. Assignation of clusters to the 2D barcoding data by different clustering methods.**

A) Manually gated 2D barcoding data. Density plot of 2D barcoding data shown in Figure 3a (main text). An expert experimentalist recognized 20 clusters and manually gated them (purple lines). White labels are attached automatically to every gated cluster by flowJo. The value on each label is the percentage of the total number of points falling within the respective gate. An integer code number is assigned to each gated cluster, from 1 to 20. The clusters in the upper row are numbered from left to right from 1 to 5, in the second row from 6 to 10, etc. B) Assignations by Misty Mountain clustering (see figure legends of Figure 3b-main text). C) Assignations by FLAME in the case of optimal cluster number: 12. Colored numbers on the right hand side are the code numbers assigned by FLAME to each cluster. Points of similar color represent a cluster according to the FLAME clustering. D) Assignations by flowClust in the case of optimal cluster number: 15. Colored numbers on the right hand side are the code numbers assigned by flowClust to each cluster. Points of similar color represent a cluster according to the flowClust clustering. E) Assignations by flowMerge at the optimal cluster number: 11. Colored numbers on the right hand side are the code numbers assigned by flowMerge to each cluster. Points of similar color represent a cluster according to the flowMerge clustering. F) Result of the flowJo clustering. Rectangular regions of the 2D barcoding plot, highlighted by different colors, signify the locations of the flowJo assigned clusters. The table to the right lists the cluster characteristics. The code number of each cluster and the respective color code is shown in the 4^th^ and 2^nd^ column of the table. The 3^rd^ column lists the number of cells belonging to each cluster. The last two columns characterize the y and x coordinate of each cluster as follows: (-) 1^st^ quarter, (+) 2^nd^ quarter, (++) 3^rd^ quarter, (+++) 4^th^ quarter of the plot.

***2.1.2 Misty Mountain clustering***

The 2D barcoding data has been analyzed by Misty Mountain clustering. The details of the analysis are described in the Results and Discussion (main text). Figure AF1b (a reproduction of Figure 3b) shows the result of the Misty Mointain clustering. In Table AF1 (column 2), the result of Misty Mountain clustering is compared with the result of manual gating. There is one-to-one correspondence between these two clustering.

At the bottom of each column of Table AF1 two parameters are listed: sensitivity and specificity. These parameters, defined in the footnotes to the table, characterize the accuracy of the respective clustering. 100% sensitivity and specificity can be attained only in the case of one to one correspondence between the manually gated and assigned clusters. Based on this standard the accuracy of Misty Mountain clustering is 100%.

The 2D barcoding data analyzed by Misty Mountain algorithm at Figure 3 (main text) contains 853,674 data points. Based on a pilot run with FLAME (at cluster number 12) the run time of the complete analysis was estimated to be more than 12 days. In order to decrease the runtime we reduced the 2D barcoding data set to 180,924 data points. In the rest of the section this reduced set of 2D barcoding data will be analyzed by different clustering methods (FLAME, flowClust, flowMerge, flowJo) and the results will be compared to the manual gating.

**Table AF1** – Clusters assigned by different methods to the manually gated 2D barcoding data and the accuracy of the clustering methods

| Manual gating | Misty Mountain | FLAME | | flowClust | | flowMerge | flowJo |
| --- | --- | --- | --- | --- | --- | --- | --- |
|  |  | Optimal clust#:12 | Optimal clust#:24 | Optimal clust#:15 | Optimal clust#:22 | Optimal clust#11 |  |
| 1 | 1 | 7 | 4 | 7 | 17 | 10 | 6 |
| 2 | 2 | 4 | 17 | 6 | 3 | 6 | $\underline{8},\bar{9}$ |
| 3 | 3 | $\underline{8},\bar{5}$ | 16 | 10 | 7 | 9 | $\bar{9}$ |
| 4 | 4 | $\overline{5}$ | $\underline{1},\underline{10},\underline{8}$ | 14 | 15 | 4 | 12 |
| 5 | 5 | $\overline{5}$ | 14 | 13 | 4 | $\overline{7}$ | 13 |
| 6 | 6 | $\bar{1}\bar{0}$ | 12 | 3 | 19 | 3 | 19 |
| 7 | 7 | $\bar{1}\bar{2}$ | $\underline{5},\underline{15}$ | 1 | $\underline{10},\underline{1}$ | $\overline{1}$ | $\underline{5},\bar{1}$ |
| 8 | 8 | $\bar{3},\overline{11}$ | $\underline{7},\underline{11}$ | 12 | 18 | $\overline{1}$ | $\bar{1},\overline{10}$ |
| 9 | 9 | $\overline{11}$ | $\underline{2},\underline{21}$ | 11 | 22 | $\overline{7}$ | $\overline{10},\overline{15}$ |
| 10 | 10 | $\overline{11}$ | $\underline{6},\overline{13}$ | $\underline{2},\overline{15}$ | 6 | $\underline{11},\overline{5}$ | $\overline{15}$ |
| 11 | 11 | $\overline{12}$ | 24 | $\bar{8}$ | 5 | $\bar{8}$ | $\overline{16}$ |
| 12 | 12 | $\overline{12}$ | $\bar{3}$ | $\bar{8}$ | 12 | $\bar{8}$ | $\overline{16},\overline{18}$ |
| 13 | 13 | $\bar{3}$ | $\bar{3}$ | $\bar{8}$ | $\overline{14}$ | $\bar{8}$ | $\overline{18},\underline{17},\bar{1}$ |
| 14 | 14 | $\overline{11}$ | 20 | $\bar{8}$ | $\overline{14}$ | $\bar{8}$ | $\bar{1},\overline{10}$ |
| 15 | 15 | $\overline{11}$ | $\bar{3},\overline{13}$ | $\overline{15}$ | $\overline{14}$ | $\overline{5}$ | $\overline{10},\overline{15}$ |
| 16 | 16 | 2 | 23 | $\underline{5},\bar{9}$ | 13 | $\overline{2}$ | 2 |
| 17 | 17 | 1 | 8 | $\bar{9}$ | $\underline{20},\underline{8}$ | $\overline{2}$ | 3 |
| 18 | 18 | $\underline{9},\bar{6}$ | 9 | $\bar{9}$ | $\underline{16},\overline{9}$ | $\overline{2}$ | 7 |
| 19 | 19 | $\bar{6}$ | 22 | $\bar{9},\bar{4}$ | $\underline{11},\overline{9}$ | $\overline{2}$ | 11 |
| 20 | 20 | $\bar{6}$ | 19 | $\bar{4}$ | $\underline{21},\underline{2}$ | $\overline{2}$ | 14 |
| sensitivity | 20/20 | 4/20 | 12/20 | 9/20 | 12/20 | 5/20 | 9/20 |
| specificity | 20/20 | 4/12 | 12/24 | 9/15 | 12/22 | 5/11 | 9/19 |

Simple number: code number of an assigned cluster belonging to one and only one gated cluster

Underscored number: more than one assigned cluster belong to the gated cluster

Overscored number: more than one gated cluster belong to the assigned cluster

sensitivity = (# of correctly assigned clusters)/(# of clusters in gold standard)

specificity = (# of correctly assigned clusters)/(total # of assigned clusters)

Gold standards were independent expert manual clustering for experimental data and specified clusters for simulated data.

***2.1.3 Clustering by FLAME mixture model***

Analyzing the 2D barcoding data by FLAME (version 4) we used the default parameters and selected t-distribution. The analysis was run for cluster numbers from 12 to 24 and it took 14 hours and 57 minutes. The optimal cluster number was selected by Bayesian Information Criterion (BIC), Akaike Information Criterion (AIC) and Scale-free Weighted Ratio (SWR). Table AF2 lists the calculated BIC, AIC and SWR parameter values at each cluster number. Based on these parameter values an optimal cluster number was given by FLAME (see last row in Table AF2).

**Table AF2. SWR, BIC and AIC values calculated**

at different cluster numbers by FLAME

| Cluster number (k) | SWR | BIC | AIC |
| --- | --- | --- | --- |
| 12 | 0.217507 | 5457067 | 5456228 |
| 13 | 0.211501 | 5433927 | 5433017 |
| 14 | 0.19881 | 5413811 | 5412831 |
| 15 | 0.180444 | 5405502 | 5404451 |
| 16 | 0.169907 | 5396301 | 5395180 |
| 17 | 0.169542 | 5395587 | 5394395 |
| 18 | 0.169141 | 5395242 | 5393978 |
| 19 | 0.167786 | 5394987 | 5393653 |
| 20 | 0.165009 | 5394973 | 5393568 |
| 21 | 0.164713 | 5393656 | 5392181 |
| 22 | 0.162179 | 5394321 | 5392775 |
| 23 | 0.162441 | 5393205 | 5391588 |
| 24 | 0.1574 | 5392127 | 5390439 |
| optimal | 24 | 12 | 12 |

Different criterions result in vastly different optimal cluster numbers: 12 and 24.

These optimal cluster numbers are equal with the lower and upper limit of the interval of the cluster numbers provided by us as input parameters. Figure AF1c shows the 12 optimal clusters that are assigned by FLAME to the 2D barcoding data.

The comparison between Figure AF1a and Figure AF1c shows that there is no one to one correspondence between the manual gating and FLAME clustering. Table AF1 (columns 3, 4) shows which FLAME assigned cluster corresponds to a gated cluster. FLAME sometimes assigns one cluster to multiple gated clusters (see overscored code#’s in Table AF1), sometimes it assigns more than one clusters to one gated cluster (see underscored code#’s in Table AF1). Table AF1 was constructed by comparing one by one the FLAME assigned clusters with the plot of the manually gated clusters. In the case of optimal cluster number 12 the sensitivity and specificity of FLAME clustering were 20% and 33%, respectively, while in the case of optimal cluster number 24 they were 60% and 50%.

***2.1.4 Clustering by flowClust mixture model***

Analyzing the 2D barcoding data by flowClust we used the default parameter values of the program. The analysis was run for cluster numbers from 12 to 23. The total run time was 14 hours 20 minutes. The optimal cluster number was selected by BIC and Integrated Completed Likelihood (ICL). Figure AF2 plots BIC and ICL against the cluster number. Each parameter has two maxima: at the same cluster numbers: 15 and 22.


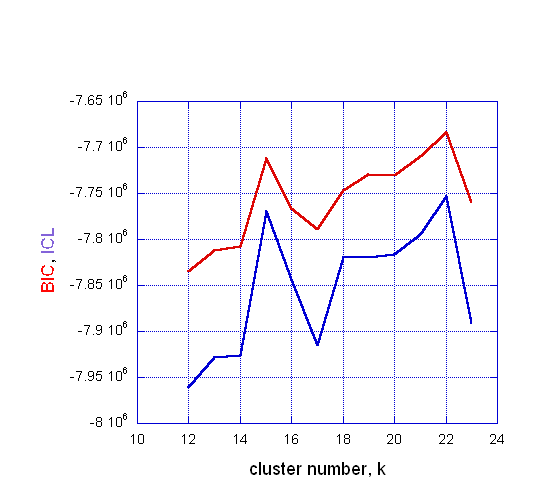


**Figure AF2. BIC and ILC parameters calculated at different cluster numbers by flowClust.** 2D barcoding data are analyzed by flowClust. The calculated BIC and ILC values are connected by red and blue lines, respectively.

Thus flowClust analysis suggests that the 2D barcoding data contains either 15 or 22 clusters.

The comparison between Figure AF1a and Figure AF1d shows that there is no one to one correspondence between the manual gating and flowClust clustering. In Table AF1 (columns 5, 6) we compared the two clustering results of flowClust with the result of the manual gating. In the case of optimal cluster number 15 the sensitivity and specificity of flowClust clustering were 45% and 60%, respectively, while in the case of optimal cluster number 22 they were 60% and 55%.

***2.1.5 Clustering by flowMerge mixture model***

Analyzing the 2D barcoding data set by flowMerge we used the following parameters of the program: randomStart=50; B=1000; B.init=100; nu.est=1; tol.init=0.01; tol=1e-5. The analysis was run for cluster numbers from 1 to 25 and it took 35 houurs. The optimal cluster number, k=11, was selected by analyzing the entropy of clustering vs. the cumulative number of merged observations plot. In Table AF3 BIC, ICL, entropy, cumulative number and CPU time are listed for each cluster number.

**Table AF3. BIC, ICL, entropy and cumulative numbers calculated**

**at different cluster numbers by flowMerge**

| Cluster number (k) | FlowClust BIC | FlowClust ICL | FlowMerge Entropy | Cumulative number | Time in seconds for flow.clust |
| --- | --- | --- | --- | --- | --- |
| 1 | -5877244.509 | -5877244.509 | 1.90E-12 | 0 | 90.39 |
| 2 | -5652061.06 | -5652787.468 | 369.9414483 | 1.7e5 | 406.63 |
| 3 | -5651980.704 | -5721354.957 | 1133.75911 | 3.2e5 | 587.065 |
| 4 | -5648224.48 | -5723340.915 | 1941.804844 | 4.6e5 | 814.086 |
| 5 | -5577710.891 | -5641434.378 | 3641.284559 | 6.0e5 | 1083.415 |
| 6 | -5631266.692 | -5697698.168 | 5516.26827 | 6.8e5 | 1822.558 |
| 7 | -5611840.883 | -5703568.261 | 6881.04523 | 6.8e5 | 1878.068 |
| 8 | -5611982.696 | -5746273.444 | 8388.290149 | 7.5e5 | 2178.561 |
| 9 | -5611872.927 | -5750799.665 | 12382.65095 | 8.0e5 | 2436.518 |
| 10 | -5571767.194 | -5719244.401 | 16873.33626 | 8.6e5 | 2530.693 |
| 11 | -5528143.527 | -5604567.235 | 23799.81828 | 9.1e5 | 3701.406 |
| 12 | -5525387.122 | -5621668.965 | 32485.31247 | 9.5e5 | 4633.517 |
| 13 | -5586988.845 | -5652703.824 | 45404.44642 | 10.0e5 | 4218.136 |
| 14 | -5517507.576 | -5618773.751 | 58520.93387 | 10.3e5 | 5719.841 |
| 15 | -5539201.782 | -5722381.151 | 71967.4529 | 10.6e5 | 5374.709 |
| 16 | -5511965.961 | -5583061.212 | 87583.57178 | 11.0e5 | 7006.992 |
| 17 | -5499990.8 | -5598837.396 | 109165.2082 | 11.4e5 | 6713.536 |
| 18 | -5498911.927 | -5596521.015 | 136946.8861 | 11.8e5 | 6477.986 |
| 19 | -5420264.145 | -5536108.544 | 167128.1397 | 12.0e5 | 10204.62 |
| 20 | -5483380.696 | -5596953.384 | NA | NA | 8471.163 |
| 21 | -5501172.401 | -5618854.03 | NA | NA | 7596.918 |
| 22 | -5539492.892 | -5750326.382 | NA | NA | 6948.014 |
| 23 | -5452366.84 | -5562271.826 | NA | NA | 11308.73 |
| 24 | -5448625.429 | -5527950.964 | NA | NA | 11929.35 |
| 25 | -5467644.852 | -5532911.499 | NA | NA | 13529.28 |

The comparison between Figure AF1a and Figure AF1e shows that there is no one to one correspondence between the manual gating and flowMerge clustering. In Table AF1 (column 7) we compared the clustering result of flowMerge with the result of the manual gating. The sensitivity and specificity of flowMerge clustering were 25% and 45%, respectively.

***2.1.6 Clustering by using flowJo flow cytometer’s clustering program***

The comparison between Figure AF1a and Figure AF1f shows that there is no one to one correspondence between the manual gating and flowJo clustering. In Table AF1 (column 8) flowJo clustering result is compared with the manual gating. The sensitivity and specificity of flowJo clustering were 45% and 47%, respectively.

***2.1.7 Clustering 2D barcoding data. Concluding remarks.***

The above group of clustering is very special because it can be compared with the standard result of manual clustering. These comparisons resulted in the following four observations:

1) There are no one-to-one correspondence between the manual gating and the results of state of the art clustering methods: FLAME, flowClust, flowMerge and flowJo. These methods sometimes assign one cluster to multiple gated clusters, sometimes assign more than one clusters to one gated cluster. The accuracy of each clustering has been characterized by two parameters: sensitivity and specificity. In the case of the above methods the accuracy of the clustering were between 20-60%, while it was 100% for Misty Mountain clustering.

2) The computation times for FLAME, flowClust and flowMerge were several orders of magnitude longer than for flowJo and Misty Mountain. In the case of Misty Mountain the runtime increases linearly with the number of data points, while in the case of FLAME the increase is superlinear.

3) An interval of possible cluster numbers should be provided as input for both FLAME and flowClust, and then the optimal cluster number is calculated by these clustering methods. FLAME has five, flowClust has two criteria for selecting the optimal cluster number. However, different criteria frequently result in different optimal cluster numbers or one criterion suggests more than one optimal cluster number.

4) In the case of FLAME frequently the optimal cluster number is equal with either the lower or upper limit of the interval of the cluster numbers provided by the user as input parameters.

The first observation could be made only by analyzing a standard data set (manually gated data). However, we can make observation 2-4 when analyzing non-standard data sets too (see below).

**2.2 Clustering 3D rituximab data by different methods**

***2.2.1 Rituximab data***

In this Section we analyze a 3D flow cytometry dataset from a drug screening

project to identify agents that would enhance the anti-lymphoma activity

of Rituximab, a therapeutic monoclonal antibody. This data set, containing only 1545 points, is an example of low-density populations. The FL1.H/FL3.H projection of the same data set served as a test example for flowClust (http://www.bioconductor.org/packages/2.2/bioc/vignettes/flowClust/inst/doc/flowClust.pdf). Figure AF3 shows two 2D projections of the data.

**
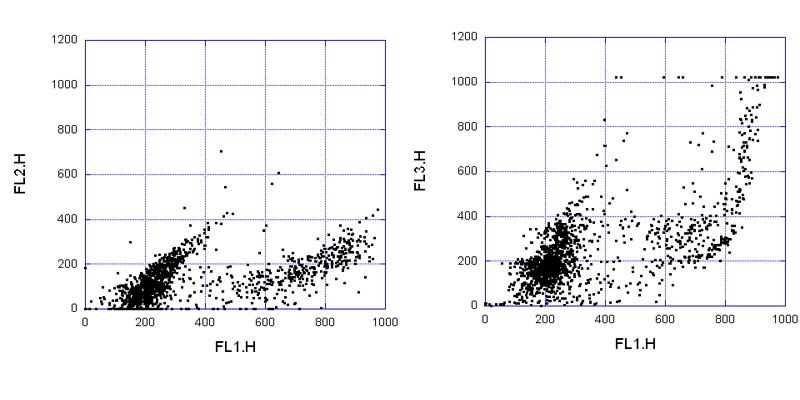
**

**Figure AF3. 2D projections of 3D rituximab FCM data set.**

***2.2.2 Misty Mountain clustering***

Misty Mountain algorithm assigned 2 clusters to the 3D rituximab data set within 0.3 sec. The analyzed histogram of the simulated data contained 8^3^ bins. The clusters contain 82.4% of the analyzed data points. Table AF4 lists the characteristics of the clusters assigned by Misty Mountain.

**Table AF4. Characteristics of the clusters assigned by Misty Mountain to**

the 3D rituximab data

| Code # | $L_{p}$ | $L_{s}$ | $C$ | $f$ |
| --- | --- | --- | --- | --- |
| 1 | 292 | 6 | 1080 | 0.979 |
| 2 | 32 | 6 | 193 | 0.813 |

(see legends to Table 1 – main text)

Figure AF4 shows the two clusters that are assigned by Misty Mountain to the 3D rituximab data.


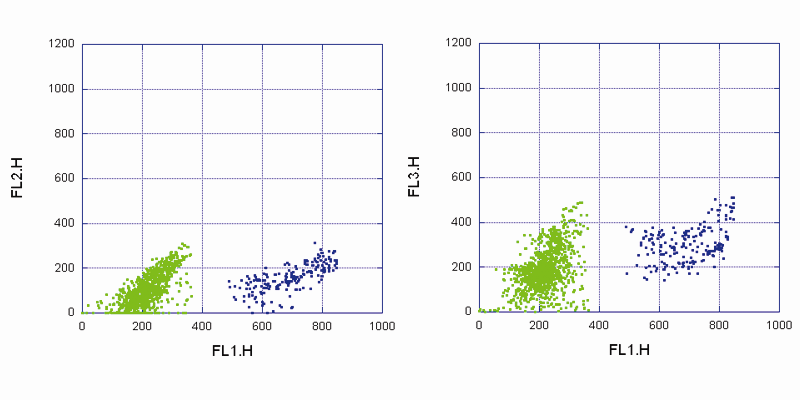


**Figure AF4. Misty Mountain clustering of 3D rituximab data.** Misty Mountain assigned two clusters to the data set. In the 2D projections of the result elements of cluster 1 and 2 are color coded by green and blue points, respectively.

***2.2.3 Clustering by FLAME mixture model***

Analyzing the 3D rituximab data set by FLAME (version 4) we used the default parameters and selected t-distribution. The analysis was run for cluster numbers from 1 to 4 and it took 10 seconds. The optimal cluster number was selected by BIC, AIC and SWR. Table AF5 lists the calculated BIC, AIC and SWR parameter values at each cluster number. Based on these parameter values an optimal cluster number was given by FLAME (see last row in Table AF5).

**Table AF5. SWR, BIC and AIC values calculated**

**at different cluster numbers by FLAME**

| cluster number (k) | SWR | BIC | AIC |
| --- | --- | --- | --- |
| 1 | Inf | 57993.7 | 57940.28 |
| 2 | 0.674273 | 54863.98 | 54751.79 |
| 3 | 0.405454 | 54608.03 | 54437.06 |
| 4 | 0.419419 | 54351.12 | 54121.38 |
| optimal | 3 | 1 | 1 |

Figure AF5 shows the 3 optimal clusters that are assigned by FLAME to the 3D rituximab data.


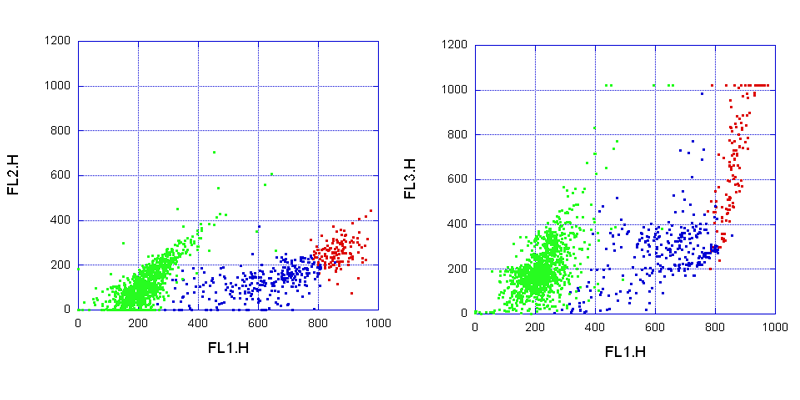


**Figure AF5. FLAME clustering of 3D rituximab data.** By using Scale-free Weighted Ratio (SWR) FLAME assigned 3 clusters to the rituximab data set. In the 2D projections of the result elements of cluster 1, 2 and 3 are color coded by green, red and blue points, respectively.

Based on BIC and AIC criterions only one optimal cluster was assigned to the rituximab data. The 2D projections of this clustering result are similar to Figure AF3 where black dots represent the elements of the cluster.

***2.2.4 Clustering by flowClust mixture model***

Analyzing the 3D rituximab data set by flowClust we used the default parameters of the program. The analysis was run for cluster numbers from 1 to 10 and it took 43 seconds. The optimal cluster number was selected by BIC and ICL. Table AF6 lists BIC and ICL for each cluster number. BIC and ICL has maximum at cluster number 7 and 2, respectively, i.e. the two criterions result in different optimal cluster numbers, 7 and 2. When the maximum of the BIC curve is rather weak flowClust suggests to choose the optimal cluster number, where the BIC curve levels off. In our case it levels off at about cluster number 4.

**Table AF6. BIC, ICL values calculated**

**at different cluster numbers by flowClust**

| Cluster number (k) | BIC | ICL | Time in seconds |
| --- | --- | --- | --- |
| 1 | -50151.9 | -50151.9 | 0.207 |
| 2 | -47887.1 | -47928.8 | 1.168 |
| 3 | -47668.5 | -48334.5 | 3.274 |
| 4 | -47503.6 | -48274.2 | 3.55 |
| 5 | -47570.3 | -48474 | 3.393 |
| 6 | -47493.6 | -48423.9 | 4.858 |
| 7 | -47412.2 | -48594.8 | 5.819 |
| 8 | -47427.9 | -48854.2 | 5.99 |
| 9 | -47481.5 | -49248.6 | 7.794 |
| 10 | -47456.7 | -48951.2 | 7.347 |
| optimal | 7 or 4 | 2 |  |

Figure AF6 shows the optimal clusters assigned by flowClust to the 3D rituximab data.


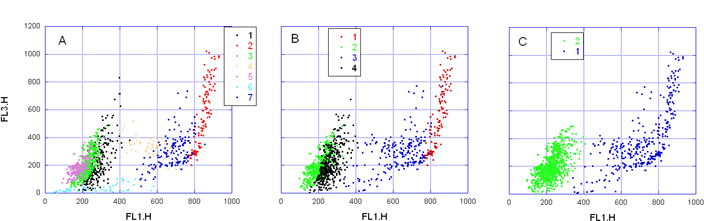


**Figure AF6. Clustering result of flowClust on 3D rituximab data set.** a),b) and c) Case of optimal cluster number 7, 4 and 2, respectively. Colored numbers in the upper part of the sub-figures are the code numbers assigned by flowClust to each cluster. Points of similar color represent a cluster according to the flowClust clustering.

***2.2.5 Clustering by flowMerge mixture model***

Analyzing the 3D rituximab data set by flowMerge we used the following parameters of the program: randomStart=50; B=1000; B.init=100; nu.est=1; tol.init=0.01; tol=1e-5. The analysis was run for cluster numbers from 1 to 10 and it took 124 seconds. The optimal cluster number, k=3, was selected by analyzing the entropy of clustering vs. the cumulative number of merged observations plot. In Table AF7 BIC, ICL, entropy, cumulative number and CPU time are listed for each cluster number.

**Table AF7. BIC, ICL, entropy and cumulative numbers calculated**

at different cluster numbers by flowMerge

| Cluster number (k) | FlowClust BIC | Flowclust ICL | FlowMerge Entropy | Cumulative number | Time in seconds for flowClust |
| --- | --- | --- | --- | --- | --- |
| 1 | -50079 | -50079 | 8.33E-15 | 0 | 0.317 |
| 2 | -47875 | -47906 | 114.82 | 1300 | 4.617 |
| 3 | -47771 | -48223 | 238.06 | 1650 | 6.347 |
| 4 | -47473 | -48195 | 442.12 | 1950 | 9.655 |
| 5 | -47418 | -48198 | 949.82 | 2900 | 13.375 |
| 6 | -47347 | -48407 | 1528.7 | 3550 | 14.919 |
| 7 | -47405 | -48706 | NA | NA | 13.739 |
| 8 | -47362 | -48566 | NA | NA | 18.893 |
| 9 | -47434 | -49012 | NA | NA | 18.586 |
| 10 | -47425 | -48769 | NA | NA | 23.598 |

Figure AF7 shows the optimal clusters assigned by flowMerge to the 3D rituximab data.


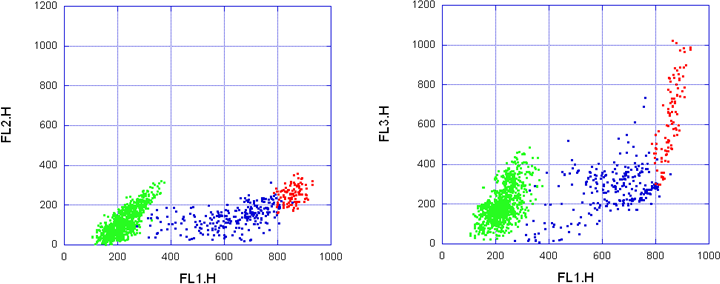


**Figure AF7. Clustering result of flowMerge on 3D rituximab data set.** By analyzing the entropy of clustering flowMerge assigned 3 clusters to the rituximab data set. In the 2D projections of the result elements of cluster 1, 2 and 3 are color coded by green, blue and red points, respectively.

***2.2.5 Clustering 3D rituximab data. Concluding remarks***

In Table AF8 the centers of the clusters assigned by FLAME, flowClust, flowMerge and Misty Mountain clustering are listed. The i-th coordinate of the center of each cluster was calculated by averaging the i-th coordinates of the *C* cluster elements: $X_{i}^{center}=\sum_{j=1}^{C} X_{i}(j)/C$.

**Table AF8. Comparing cluster centers assigned by**

different clustering methods to rituximab data.

| Cluster color code | green | blue | red | black |
| --- | --- | --- | --- | --- |
| **FLAME clustering** |  |  |  |  |
| code # | 1 | 3 | 2 |  |
| Cluster size, *C* | 1132 | 278 | 135 |  |
| $X_{1}^{center}$ | 221.47 | 619.11 | 864.03 |  |
| $X_{2}^{center}$ | 110.43 | 127.36 | 258.3 |  |
| $X_{3}^{center}$ | 203.74 | 273.38 | 685.27 |  |
| **flowClust clustering**  **(case of 2 clusters)** |  |  |  |  |
| code # | 2 | 1 |  |  |
| Cluster size, *C* | 915 | 342 |  |  |
| $X_{1}^{center}$ | 225.4 | 713.1 |  |  |
| $X_{2}^{center}$ | 118.7 | 175.9 |  |  |
| $X_{3}^{center}$ | 198.5 | 391.8 |  |  |
| **flowClust clustering**  **(case of 4 clusters)** |  |  |  |  |
| code # | 2 | 3 | 1 | 4 |
| Cluster size, *C* | 547 | 217 | 127 | 387 |
| $X_{1}^{center}$ | 201.57 | 633.1 | 843.07 | 263.77 |
| $X_{2}^{center}$ | 75 | 135.75 | 243.97 | 185.4 |
| $X_{3}^{center}$ | 195 | 291.8 | 556.95 | 210 |
| **flowMerge clustering** |  |  |  |  |
| code # | 1 | 2 | 3 |  |
| Cluster size, *C* | 869 | 252 | 103 |  |
| $X_{1}^{center}$ | 224.8 | 631.94 | 854.34 |  |
| $X_{2}^{center}$ | 117.74 | 140.12 | 252.29 |  |
| $X_{3}^{center}$ | 199.41 | 272.98 | 618.93 |  |
| **Misty Mountain clustering** | |  |  |  |
| code # | 1 | 2 |  |  |
| Cluster size, *C* | 1080 | 193 |  |  |
| $X_{1}^{center}$ | 215.81 | 694.54 |  |  |
| $X_{2}^{center}$ | 100.97 | 158.77 |  |  |
| $X_{3}^{center}$ | 189.87 | 305.33 |  |  |

Based on the 2D projections and center coordinates of the assigned clusters one can compare the results of different clustering methods. Characteristics of corresponding clusters, found by different clustering methods, are arranged in the same column of Table AF8. Thus the result of Misty Mountain clustering of 3D rituximab data is most similar to the ICL-based flowClust clustering result. Both of these methods find two clusters with rather similar cluster center locations. In those cases when the optimal cluster number is larger than 2 the originally found two clusters are dissected to smaller clusters. Table AF8 does not contain the centers of 7 clusters that were assigned by flowClust (see Figure AF6a). This clustering dissects the clusters shown in Figure AF6c, i.e. clusters with code numbers 1, 3, 5 and 6 are all part of the green cluster in Figure AF6c.

**2.3 Clustering 4D GvHD data by different methods**

***2.3.1 GvHD data***

One of the graft-versus-host disease data sets (GVHD2.iso, Folder E#21 H06) is shown in Figure 4 (main text). This data set is an example for overlapping populations.

***2.3.2 Misty Mountain clustering***

The GvHD data set was analyzed by Misty Mountain algorithm (see Figure 5 in main text) and it assigned 6 clusters to the 4D GvHD data set within 0.8 second. Table 4 (main text) lists the characteristics of the clusters assigned by Misty Mountain.

***2.3.3 Clustering by FLAME mixture model***

Analyzing the GvHD data set by FLAME (version 4) we used the default parameters and selected t-distribution. The analysis was run for cluster numbers from 3 to 8 and it took 6 minutes. The optimal cluster number was selected by BIC, AIC and SWR. Table AF9 lists the calculated BIC, AIC and SWR parameter values at each cluster number. Based on these parameter values an optimal cluster number was given by FLAME (see last row in Table AF9).

**Table AF9. SWR, BIC and AIC values calculated**

**at different cluster numbers by FLAME**

| Cluster number (k) | SWR | BIC | AIC |
| --- | --- | --- | --- |
| 3 | 0.628365 | 864208.8 | 863838.2 |
| 4 | 0.505792 | 856417.7 | 855921 |
| 5 | 0.512694 | 853966.5 | 853343.6 |
| 6 | 0.502394 | 851494.9 | 850745.8 |
| 7 | 0.518032 | 850712.4 | 849837.2 |
| 8 | 0.467142 | 849621.2 | 848619.8 |
| optimal | 8 | 3 | 3 |

The 2D projections of the clustering results for cluster number 3 and 8 are shown in Figure AF8 and AF9, respectively.

**
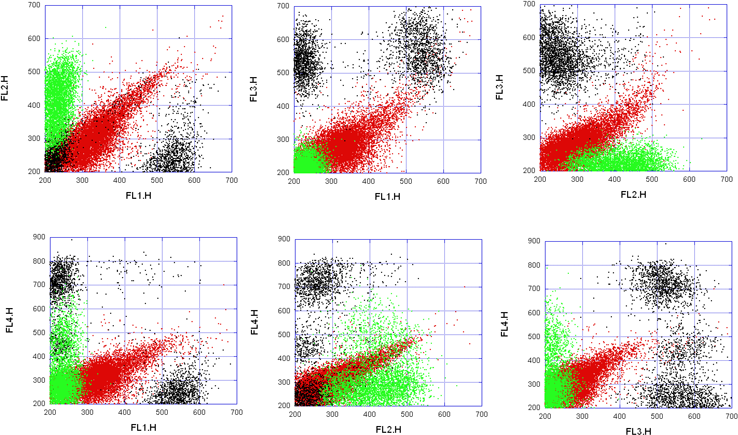
**

**Figure AF8. Three clusters assigned by FLAME to the 4D GvHD data set.**

2D projections of the 4D clustering result. Code numbers of clusters assigned by FLAME algorithm: 1 (red); 2 (black); 3 (green).

**
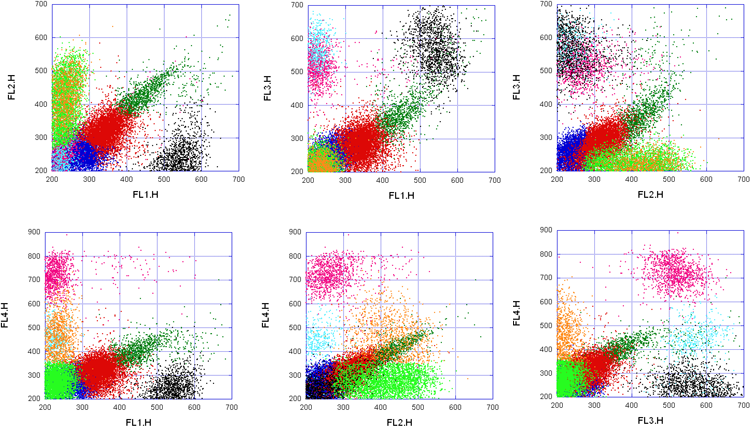
**

**Figure AF9. Eight clusters assigned by FLAME to the 4D GvHD data set.**

2D projections of the 4D clustering result. Code numbers of clusters assigned by FLAME

algorithm: 1 (blue); 2 (rose); 3 (dark green); 4(red); 5(light blue); 6(green); 7(brown); 8(black).

***2.3.4 Clustering by flowClust mixture model***

Analyzing the GvHD data set by flowClust we used the default parameters of the program. The analysis was run for cluster numbers from 1 to 10 and it took almost 8 minutes. The optimal cluster number was selected by BIC and ICL. Table AF10 lists BIC and ICL for each cluster number. BIC levels off and ICL has maximum at cluster number 4, i.e. the two criterions result in the same optimal cluster number: 4.

**Table AF10. BIC, ICL values calculated**

**at different cluster numbers by flowClust**

| Cluster number (k) | BIC | ICL | Time in seconds |
| --- | --- | --- | --- |
| 1 | -823773 | -823773 | 1.288 |
| 2 | -795566 | -796222 | 9.229 |
| 3 | -785518 | -792337 | 12.123 |
| 4 | -774578 | -776939 | 26.47 |
| 5 | -772078 | -782081 | 48.663 |
| 6 | -771801 | -787054 | 35.826 |
| 7 | -770038 | -786118 | 61.452 |
| 8 | -770300 | -787582 | 62.842 |
| 9 | -769911 | -792126 | 93.477 |
| 10 | -769542 | -793941 | 112.359 |
| optimal | 4 | 4 |  |

2D projections of the flowClust clustering result are shown in Figure AF10.

**
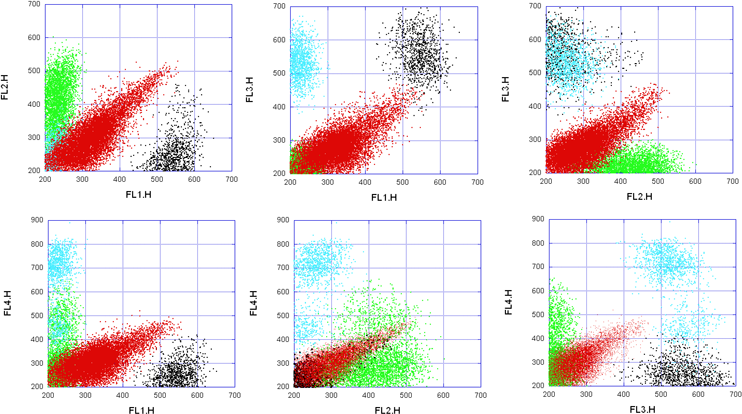
**

**Figure AF10. Four clusters assigned by flowClust to the 4D GvHD data set.**

2D projections of the 4D clustering result. Code numbers of clusters assigned by flowClust algorithm are: 1 (black); 2 (green); 3 (light blue); 4(red).

***2.3.5 Clustering by flowMerge mixture model***

Analyzing the 4D GvHD data set by flowMerge we used the following parameters of the program: randomStart=50; B=1000; B.init=100; nu.est=1; tol.init=0.01; tol=1e-5. The analysis was run for cluster numbers from 1 to 10 and it took 17 minutes. The optimal cluster number, k=5, was selected by analyzing the entropy of clustering vs. the cumulative number of merged observations plot. In Table AF11 BIC, ICL, entropy, cumulative number and CPU time are listed for each cluster number.

**Table AF11. BIC, ICL, entropy and cumulative numbers calculated**

at different cluster numbers by flowMerge

| Cluster number (k) | FlowClust BIC | FlowClust ICL | FlowMerge Entropy | Cumulative number | Time in seconds for flowClust |
| --- | --- | --- | --- | --- | --- |
| 1 | -8.14E+05 | -8.14E+05 | 1.98E-13 | 0 | 2.2 |
| 2 | -7.94E+05 | -7.95E+05 | 54.077 | 17000 | 29.31 |
| 3 | -7.85E+05 | -7.87E+05 | 207.12 | 32000 | 51.619 |
| 4 | -7.73E+05 | -7.74E+05 | 400.62 | 46000 | 74.017 |
| 5 | -7.72E+05 | -7.79E+05 | 2204.5 | 61000 | 85.445 |
| 6 | -7.69E+05 | -7.80E+05 | 7835.4 | 71000 | 111.455 |
| 7 | -7.69E+05 | -7.85E+05 | 15987 | 81000 | 116.839 |
| 8 | -7.68E+05 | -7.83E+05 | 22578 | 90000 | 180.76 |
| 9 | -7.68E+05 | -7.87E+05 | NA | NA | 170.686 |
| 10 | -7.68E+05 | -7.91E+05 | NA | NA | 210.953 |

Figure AF11 shows the optimal clusters assigned by flowMerge to the 4D GvHD data.

**
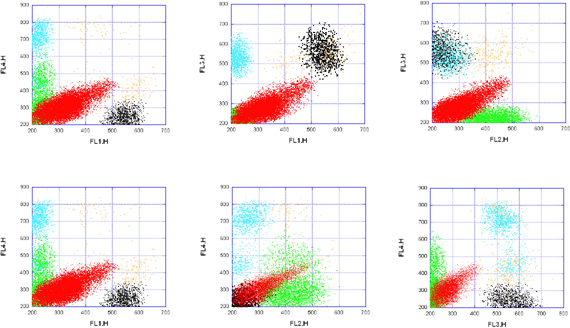
**

**Figure AF11. Five clusters assigned by flowMerge to the 4D GvHD data set.**

2D projections of the 4D clustering result. Code numbers of clusters assigned by flowMerge algorithm are: 1 (red); 2 (green); 3 (light brown); 4(black); 5(light blue).

***2.3.6 Clustering by using flowJo flow cytometer’s clustering program***

We analyzed the 4D GvHD data set by flowJo’s clustering program. The 2D projections of the clustering result and the list of the cluster characteristics are shown in Figure AF12.

**
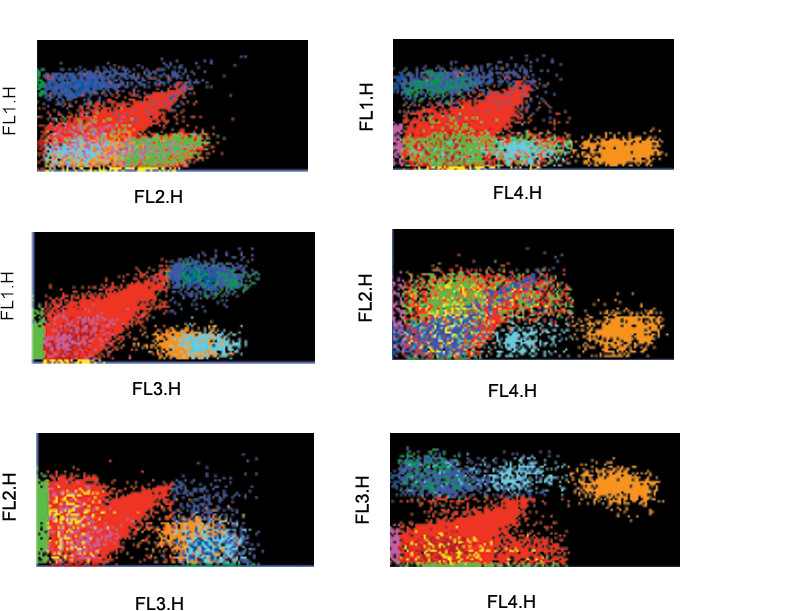
**

***
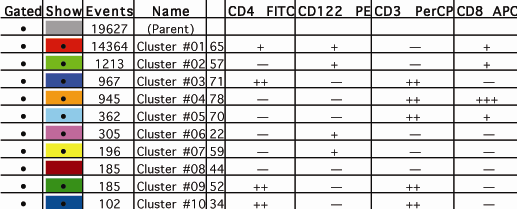
***

**Figure AF12. Result of the flowJo clustering of the 4D GvHD data.** 2D projections of the clustering result. The code number of each cluster and the respective color code is shown in the 4^th^ and 2^nd^ column of the table to the figure. The 3^rd^ column lists the number of cells belonging to each cluster. The last two columns characterize the y and x coordinate of each cluster as follows: (-) 1^st^ quarter, (+) 2^nd^ quarter, (++) 3^rd^ quarter, (+++) 4^th^ quarter of the plot.

***2.3.7 Clustering 4D GvHD data. Concluding remarks***

In Table AF12 the centers of the clusters assigned by FLAME, flowClust, flowMerge and Misty Mountain clustering are listed. The i-th coordinate of the center of each cluster was calculated by averaging the i-th coordinates of the *C* cluster elements: $X_{i}^{center}=\sum_{j=1}^{C} X_{i}(j)/C$.

**Table AF12. Comparing cluster centers assigned by**

**different clustering methods to GvHD data.**

| Cluster color code | blue | rose | dark green | red | light blue | brown | green | black | light brown |
| --- | --- | --- | --- | --- | --- | --- | --- | --- | --- |
| **FLAME clustering, 8 clusters** |  |  |  |  |  |  |  |  |  |
| code # | 1 | 2 | 3 | 4 | 5 | 7 | 6 | 8 |  |
| Cluster size, *C* | 4811 | 1150 | 1370 | 5649 | 465 | 1107 | 3755 | 1319 |  |
| $X_{1}^{center}$ | 272.3 | 243.69 | 448.66 | 345.63 | 228.4 | 238.74 | 233.25 | 539.05 |  |
| $X_{2}^{center}$ | 249.8 | 273.15 | 427.49 | 320.83 | 234.42 | 426.56 | 404.04 | 241.39 |  |
| $X_{3}^{center}$ | 252.85 | 515.76 | 378.31 | 285.38 | 572.33 | 219.34 | 220.85 | 565.49 |  |
| *X_4_^center^* | 269.9 | 718.64 | 399.3 | 316.34 | 423.34 | 466.24 | 269.91 | 244.49 |  |
| **FLAME clustering, 3 clusters** |  |  |  |  |  |  |  |  |  |
| code # |  |  |  | 1 |  |  | 3 | 2 |  |
| Cluster size, *C* |  |  |  | 11807 |  |  | 4832 | 2987 |  |
| $X_{1}^{center}$ |  |  |  | 326.46 |  |  | 234.27 | 377.71 |  |
| $X_{2}^{center}$ |  |  |  | 303.75 |  |  | 409.64 | 256.1 |  |
| $X_{3}^{center}$ |  |  |  | 281.44 |  |  | 220.3 | 547.95 |  |
| *X_4_^center^* |  |  |  | 306.34 |  |  | 315.14 | 458.06 |  |
| **flowClust clustering, 4 clusters** |  |  |  |  |  |  |  |  |  |
| code # |  |  |  | 4 | 3 |  | 2 | 1 |  |
| Cluster size, *C* |  |  |  | 10885 | 1342 |  | 4011 | 988 |  |
| $X_{1}^{center}$ |  |  |  | 328.31 | 229.24 |  | 234.77 | 545.15 |  |
| $X_{2}^{center}$ |  |  |  | 305.4 | 257.04 |  | 405.57 | 255.99 |  |
| $X_{3}^{center}$ |  |  |  | 282.35 | 531.54 |  | 224.96 | 556.7 |  |
| *X_4_^center^* |  |  |  | 308.46 | 646.63 |  | 313.04 | 257.63 |  |
| **flowMerge** |  |  |  |  |  |  |  |  |  |
| code # |  |  |  | 1 | 5 |  | 2 | 4 | 3 |
| Cluster size, *C* |  |  |  | 10463 | 1269 |  | 3432 | 860 | 230 |
| $X_{1}^{center}$ |  |  |  | 325.47 | 228.02 |  | 235.08 | 543.83 | 515.57 |
| $X_{2}^{center}$ |  |  |  | 305.05 | 256.29 |  | 419.08 | 244.58 | 424.29 |
| $X_{3}^{center}$ |  |  |  | 280.81 | 531.67 |  | 223.78 | 560.03 | 540.77 |
| *X_4_^center^* |  |  |  | 308.17 | 651.06 |  | 315.16 | 248.09 | 509.12 |
| **Misty Mountain, 6 clusters** |  |  |  |  |  |  |  |  |  |
| code # | 2 | 5 |  | 1 | 6 |  | 3 | 4 |  |
| Cluster size, *C* | 1116 | 858 |  | 1542 | 265 |  | 890 | 1011 |  |
| $X_{1}^{center}$ | 224.62 | 223.19 |  | 352.27 | 226.24 |  | 227.16 | 535.26 |  |
| $X_{2}^{center}$ | 229.65 | 257.16 |  | 324.85 | 224.59 |  | 399.45 | 228.78 |  |
| $X_{3}^{center}$ | 224.97 | 517.21 |  | 301.65 | 570.63 |  | 217.69 | 575.23 |  |
| *X_4_^center^* | 244.45 | 718.27 |  | 333.98 | 438.6 |  | 247.74 | 232.21 |  |
| **flowJo clustering, largest 5 clusters** |  |  |  |  |  |  |  |  |  |
| code # |  | 4 |  | 1 | 5 |  | 2 | 3 |  |
| flowJo color |  | (l.brown) |  | (red) | (l.blue) |  | (green) | (blue) |  |
| Cluster size, *C* |  | 945 |  | 14364 | 362 |  | 1213 | 967 |  |
| $X_{1}^{center}$ |  | - |  | + | - |  | - | ++ |  |
| $X_{2}^{center}$ |  | - |  | + | - |  | + | - |  |
| $X_{3}^{center}$ |  | ++ |  | - | ++ |  | - | ++ |  |
| *X_4_^center^* |  | +++ |  | + | + |  | + | - |  |

Based on the 2D projections and center coordinates of the assigned clusters one can compare the results of different clustering methods. Characteristics of corresponding clusters, found by different clustering methods, are arranged in the same column of Table AF12. Thus the result of Misty Mountain clustering of 4D GvHD data is most similar to the SWR-based FLAME clustering result. Every center of the Misty Mountain assigned clusters is close to a cluster center identified by FLAME. However, FLAME identifies two more clusters. These extra clusters are colored in Figure AF9 by brown and dark green and result in from the dissection of the green and red cluster in Figure AF8. FlowJo assigned even more clusters to the 4D GvHD data than FLAME. Five of these clusters (listed at the bottom of Table AF12) were comparable with clusters assigned by the Misty Mountain method. Finally we note that flowMerge identified a cluster (light brown dots in Figure AF11) that is not assigned by any other clustering methods.

**2.4 Clustering 4D OP9 data by different methods**

As in Sec.2.1 here again we are able to compare the accuracy of different clustering methods by analyzing manually gated data from OP9 cells. Model based clustering methods were designed to analyze just these types of data.

***2.4.1 OP9 data***

OP9 cells, a line of bone marrow-derived mouse stromal cells, were stained by four stains. The respective FCM data set was kindly provided by Professor Hans Snoeck (Mount Sinai School of Medicine) and is available at Additional File 8. 2D projections of the 4D data set are shown in Figure AF13.


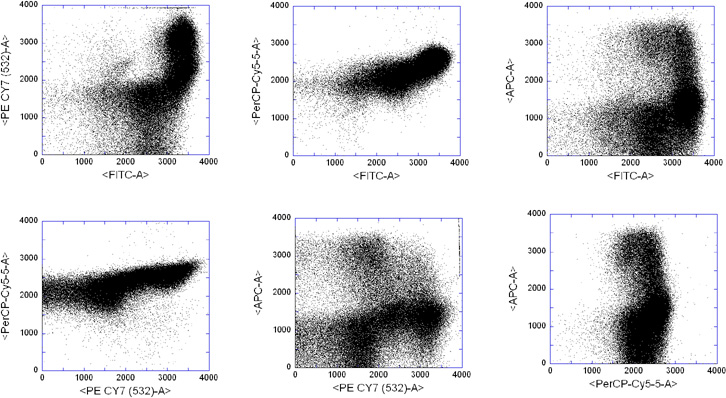


**Figure AF13. 2D projections of 4D OP9 FCM data set.** OP9 cells were stained with antibodies for 1) CD45 – FITC, 2) Gr1 – PE CY7, 3) Mac1 – PerCP-Cy5 and 4) CD19 – APC.

***2.4.2 Manual clustering***

By using flowJo two experts independently gated the OP9 FCM data set (Figure AF14). First the APC/PE CY7 2D projection was considered (Figure AF14a) and four clusters were found. Then the elements of each of these clusters were considered in the PerCP-Cy5/FITC plane (Figure AF14b-e). In this plane only one of the four clusters splitted into two clusters (Figure AF14d), while the others remained single cluster. Thus by manual gating the experts identified 5 clusters total.


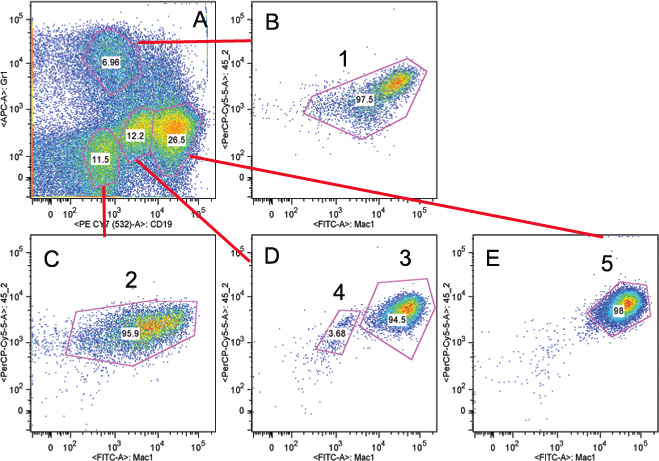


**Figure AF14. Manual gating of the 4D OP9 FCM data set.**

Density plots of the 4D OP9 dataset. a) Projection APC/PE CY7. Two experts independently gated four clusters in this projection (purple lines). b-e) Elements of each of the four clusters are projected into the PerCP-CY5/FITC plane. Each cluster and the respective projection is connected by a red line. White labels are attached automatically to every gated cluster by flowJo. The value on each label is the percentage of the total number of points falling within the respective gate. An integer code number is assigned to each gated cluster, from 1 to 5. The size and center coordinates of these clusters are listed in Table AF17.

***2.4.3 Misty Mountain clustering***

Misty Mountain algorithm assigned 5 clusters to the 4D OP9 data set within 3.6 sec. The analyzed histogram of the simulated data contained 11^4^ bins. The clusters contain 13% of the analyzed data points. Table AF13 lists the characteristics of the clusters assigned by Misty Mountain.

**Table AF13. Characteristics of the clusters assigned by Misty Mountain to**

**the 4D OP9** data

| Code # | $L_{p}$ | $L_{s}$ | $C$ | Bin# | $f$ |
| --- | --- | --- | --- | --- | --- |
| 1 | 1519 | 905 | 6937 | 6 | 0.404 |
| 2 | 455 | 310 | 1478 | 4 | 0.319 |
| 3 | 1105 | 905 | 1106 | 1 | 0.181 |
| 4 | 377 | 321 | 744 | 2 | 0.149 |
| 5 | 95 | 54 | 176 | 2 | 0.432 |

(see legends to Table 4 – main text)

The low $f$ values in Table AF13 show that the histogram peaks belonging to cluster 3, 4 are seriously overlapping with nearby peak(s). In each of these cases Misty Mountain assigns cluster to a histogram cross section that is close to the top of the respective peak and thus the number of histogram bins assigned to these seriously overlapping clusters is low.

Figure AF15 shows the 5 clusters that are assigned by Misty Mountain to the 4D OP9 data.


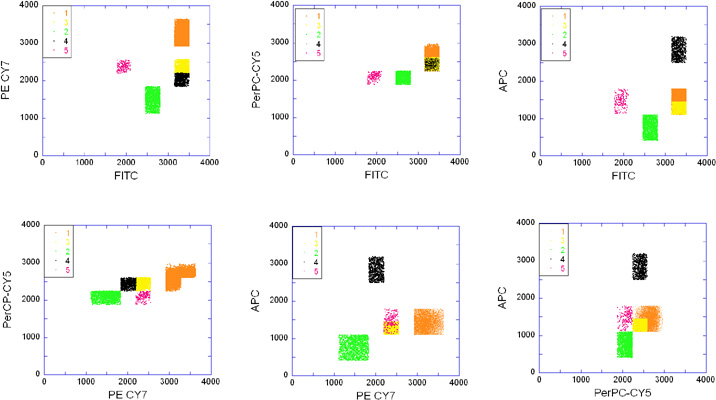


**Figure AF15. Misty Mountain clustering of 4D OP9 data.** 2D projections of the 4D clustering result. Misty Mountain assigned five clusters to the data set. Points of similar color represent a cluster according to Misty Mountain clustering. Code numbers of the clusters assigned by the Misty Mountain algorithm are: 1 (brown), 2 (yellow), 3 (green), 4 (black), 5 (pink). These code numbers and color codes can also be found in the upper left part of the sub-figures.

***2.4.4 Clustering by FLAME mixture model***

Analyzing the OP9 data set by FLAME (version 4) we used the default parameters and selected t-distribution. The analysis was run for cluster numbers from 1 to 13 and it took 3h 53 minutes. The optimal cluster number was selected by BIC, AIC and SWR criterions. Table AF14 lists the calculated BIC, AIC and SWR parameter values at each cluster number. Based on these parameter values an optimal cluster number was given by FLAME (see last row in Table AF14).

**Table AF14. SWR, BIC and AIC values calculated**

at different cluster numbers by FLAME

| Cluster number (k) | SWR | BIC | AIC |
| --- | --- | --- | --- |
| 1 | Inf | 5822259 | 5822117 |
| 2 | 0.907632 | 5742679 | 5742386 |
| 3 | 0.79435 | 5711493 | 5711049 |
| 4 | 0.745299 | 5495844 | 5495249 |
| 5 | 0.732384 | 5488466 | 5487719 |
| 6 | 0.666273 | 5478922 | 5478024 |
| 7 | 0.515816 | 5470505 | 5469456 |
| 8 | 0.489389 | 5466725 | 5465524 |
| 9 | 0.494244 | 5466028 | 5464676 |
| 10 | 0.475863 | 5463140 | 5461637 |
| 11 | 0.482081 | 5428599 | 5426945 |
| 12 | 0.459828 | 5458926 | 5457121 |
| 13 | 0.417442 | 5447392 | 5445435 |
| optimal | 13 | 1 | 1 |

The optimal cluster numbers suggested by FLAME agree with the upper and lower bond of the interval of the cluster number where the analysis was run. These optimal cluster numbers are rather different from the cluster number provided by manual gating and thus we do not further analyze the clustering results of FLAME.

***2.4.5 Clustering by flowClust mixture model***

Analyzing the OP9 data set by flowClust we used the default parameters of the program. The analysis was run for cluster numbers from 1 to 10 and it took 1h 1min. The optimal cluster number was selected by BIC and ICL. Table AF15 lists BIC and ICL for each cluster number. BIC has a faint maximum at cluster number 8 and ICL has two maxima of comparable heights at cluster numbers 2 and 4.

**Table AF15. BIC, ICL values calculated**

at different cluster numbers by flowClust

| Cluster number (k) | BIC | ICL | Time in seconds |
| --- | --- | --- | --- |
| 1 | -4872967 | -4872967 | 10.892 |
| 2 | -4810254 | -4832141 | 80.103 |
| 3 | -4801094 | -4858361 | 144.493 |
| 4 | -4783214 | -4838036 | 331.548 |
| 5 | -4774036 | -4842338 | 278.824 |
| 6 | -4768081 | -4839369 | 346.113 |
| 7 | -4764365 | -4840680 | 596.269 |
| 8 | -4767948 | -4870808 | 569.438 |
| 9 | -4762421 | -4863619 | 674.626 |
| 10 | -4762033 | -4870678 | 637.314 |
| optimal | 8 | 2 or 4 |  |

2D projections of the flowClust clustering results are shown in Figure AF16 and AF17.


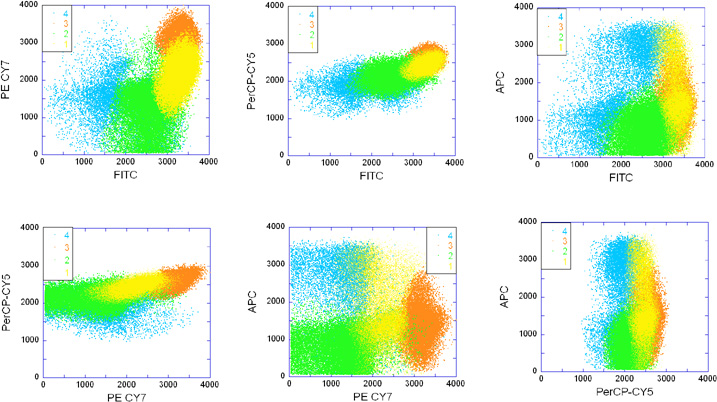


**Figure AF16. Four clusters assigned by flowClust to the 4D OP9 data set.**

2D projections of the 4D clustering result. Code numbers of clusters assigned by flowClust algorithm: 1 (yellow); 2 (green); 3 (brown); 4 (light blue).

***
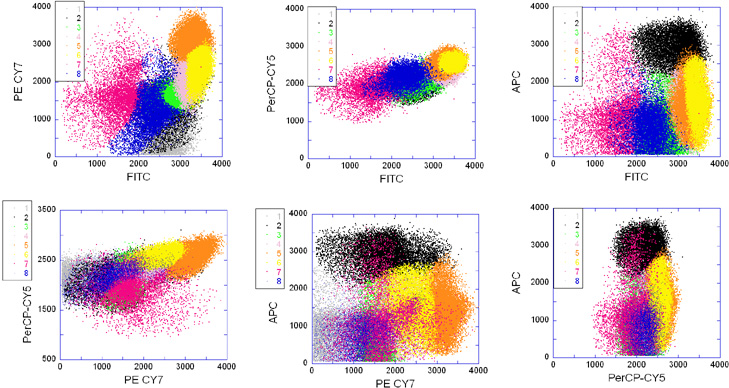
***

**Figure AF17. Eight clusters assigned by flowClust to the 4D OP9 data set.**

2D projections of the 4D clustering result. Code numbers of clusters assigned by flowClust algorithm: 1 (grey); 2 (black); 3 (green); 4 (purple); 5(brown); 6(yellow); 7(pink); 8(blue).

***2.4.6 Clustering by flowMerge mixture model***

Analyzing the 4D OP9 data set by flowMerge we used the following parameters of the program: randomStart=50; B=1000; B.init=100; nu.est=1; tol.init=0.01; tol=1e-5. The analysis was run for cluster numbers from 1 to 10 and it took 2h 20 minutes. The optimal cluster number, k=7, was selected by analyzing the entropy of clustering vs. the cumulative number of merged observations plot. In Table AF16 BIC, ICL, entropy, cumulative number and CPU time are listed for each cluster number.

**Table AF16. BIC, ICL, entropy and cumulative numbers calculated**

**at different cluster numbers by flowMerge**

| Cluster number (k) | FlowClust BIC | FlowClust  ICL | FlowMerge Entropy | Cumulative  number | Time in seconds for flowClust |
| --- | --- | --- | --- | --- | --- |
| 1 | -4872626 | -4872626 | 1.17E-12 | 0.0E0 | 9.035 |
| 2 | -4803047 | -4821066 | 6979.277953 | 0.7E5 | 260.569 |
| 3 | -4790830 | -4842451 | 12134.53506 | 1.5E5 | 417.689 |
| 4 | -4772372 | -4810064 | 25003.24129 | 2.2E5 | 635.321 |
| 5 | -4765819 | -4835201 | 39117.61756 | 2.9E5 | 753.441 |
| 6 | -4756813 | -4808160 | 53506.62272 | 3.4E5 | 908.743 |
| 7 | -4760475 | -4834112 | 68630.10328 | 4.0E5 | 1036.796 |
| 8 | -4754442 | -4827443 | 82059.32485 | 4.1E5 | 1204.346 |
| 9 | -4752723 | -4844362 | 98819.77644 | 4.5E5 | 1439.022 |
| 10 | -4749987 | -4835576 | 123479.4631 | 4.8E5 | 1836.662 |

2D projections of the flowMerge clustering result are shown in Figure AF18.

***
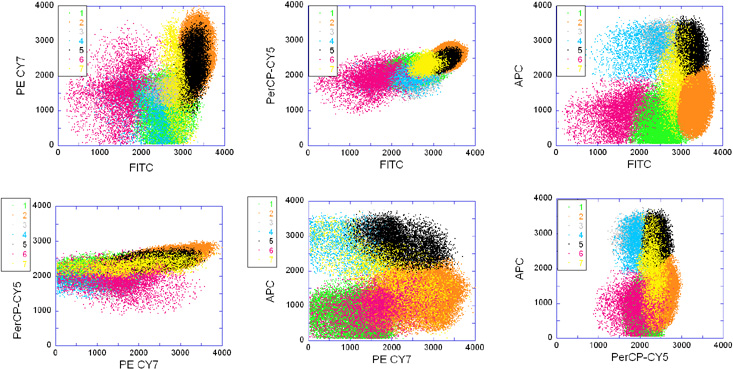
***

**Figure AF18. Seven clusters assigned by flowMerge to the 4D OP9 data set.**

2D projections of the 4D clustering result. Code numbers of clusters assigned by flowMerge algorithm: 1 (green); 2 (brown); 3 (grey); 4 (light blue); 5(black); 6(pink); 7(yellow).

***2.4.7 Clustering 4D OP9 data. Concluding remarks***

In Table AF17 the centers of the clusters assigned by manual gating, flowClust, flowMerge and Misty Mountain clustering are listed. The i-th coordinate of the center of each cluster was calculated by averaging the i-th coordinates of the *C* cluster elements: $X_{i}^{center}=\sum_{j=1}^{C} X_{i}(j)/C$. At manual gating the cluster center coordinates (cc) were transformed to channel numbers (cn) by: $cn=678\cdot\log(cc)$

**Table AF17. Comparing cluster centers assigned by**

**different clustering methods to OP9 data.**

| **Manual gating** |  |  |  |  |  |  |  |  |  |
| --- | --- | --- | --- | --- | --- | --- | --- | --- | --- |
| code # | 5 | 2 | 3 | 1 | 4 |  |  |  |  |
| Cluster size, *C* | 93100 | 10925 | 10952 | 6612 | 427 |  |  |  |  |
| $X_{1}^{center}$ | 3153.5 | 2562.5 | 3170.2 | 2907.9 | 2089.9 |  |  |  |  |
| $X_{2}^{center}$ | 2981.9 | 1858.0 | 2424.5 | 2003.6 | 2385.1 |  |  |  |  |
| $X_{3}^{center}$ | 2591.3 | 2251.2 | 2515.5 | 2341.9 | 2186.4 |  |  |  |  |
| *X_4_^center^* | 1726.6 | 1348.2 | 1720.6 | 2799.8 | 1755.2 |  |  |  |  |
| **Automatic gating** |  |  |  |  |  |  |  |  |  |
| **flowClust clustering, 4 clusters** |  |  |  |  |  |  |  |  |  |
| Cluster color code | brown | green | yellow |  |  | light-blue |  |  |  |
| code # | 3 | 2 | 1 |  |  | 4 |  |  |  |
| Cluster size, *C* | 21212 | 21457 | 24849 |  |  | 10524 |  |  |  |
| $X_{1}^{center}$ | 3326.3 | 2598.9 | 3311.6 |  |  | 1975.8 |  |  |  |
| $X_{2}^{center}$ | 3136.2 | 1301.9 | 2208.7 |  |  | 1396.6 |  |  |  |
| $X_{3}^{center}$ | 2581.7 | 2158.4 | 2456.7 |  |  | 1912.1 |  |  |  |
| *X_4_^center^* | 1377.3 | 776.04 | 1805.1 |  |  | 1971.9 |  |  |  |
| **flowClust clustering, 8 clusters** |  |  |  |  |  |  |  |  |  |
| Cluster color code | brown | green | yellow | black | pink |  | grey | purple | blue |
| code # | 5 | 3 | 6 | 2 | 7 |  | 1 | 4 | 8 |
| Cluster size, *C* | 20755 | 9451 | 11292 | 10218 | 6188 |  | 4631 | 8359 | 6480 |
| $X_{1}^{center}$ | 3323.1 | 2689.1 | 3457.8 | 2949.3 | 1530.5 |  | 2760.4 | 3207.2 | 2313.5 |
| $X_{2}^{center}$ | 3142.5 | 1593.4 | 2389.2 | 1763.9 | 1639.4 |  | 586.9 | 2090.8 | 1197.7 |
| $X_{3}^{center}$ | 2579.4 | 2112.9 | 2568.4 | 2222.9 | 1898.1 |  | 2210.8 | 2348 | 2182.6 |
| *X_4_^center^* | 1386.7 | 787.7 | 1504 | 2927.9 | 1111.6 |  | 919.8 | 1275 | 801.2 |
| **flowMerge** |  |  |  |  |  |  |  |  |  |
| Cluster color code | brown | green | yellow | black | pink | light blue | grey |  |  |
| code # | 2 | 1 | 7 | 5 | 6 | 4 | 3 |  |  |
| Cluster size, *C* | 36375 | 17405 | 2904 | 7546 | 5083 | 2465 | 2442 |  |  |
| $X_{1}^{center}$ | 3344.4 | 2541.3 | 2833.1 | 3240.2 | 1498.5 | 2122.4 | 2616.3 |  |  |
| $X_{2}^{center}$ | 2712.3 | 1248.6 | 1878.1 | 2240.7 | 1570.5 | 994.23 | 1585.5 |  |  |
| $X_{3}^{center}$ | 2534.1 | 2137.2 | 2312.4 | 2420.6 | 1887.5 | 1912.2 | 1941.6 |  |  |
| *X_4_^center^* | 1324.2 | 765.43 | 2000.0 | 2775.4 | 950.77 | 2748.1 | 3037.1 |  |  |
| **Misty Mountain clusters** |  |  |  |  |  |  |  |  |  |
| Cluster color code | brown | green | yellow | black | pink |  |  |  |  |
| code # | 1 | 2 | 3 | 4 | 5 |  |  |  |  |
| Cluster size, *C* | 6937 | 1478 | 1106 | 744 | 176 |  |  |  |  |
| $X_{1}^{center}$ | 3350.3 | 2637.7 | 3353.0 | 3309.4 | 1934.9 |  |  |  |  |
| $X_{2}^{center}$ | 3198.2 | 1506.0 | 2362.9 | 2022.8 | 2367.6 |  |  |  |  |
| $X_{3}^{center}$ | 2626.6 | 2085.0 | 2456.4 | 2427.3 | 2086.3 |  |  |  |  |
| *X_4_^center^* | 1426.6 | 760.9 | 1289.4 | 2835.6 | 1468.0 |  |  |  |  |

Based on the 2D projections and the center coordinates of the assigned clusters one can compare the results of different clustering methods. We consider the result of the manual clustering of OP9 data as our gold standard and based on the cluster correspondences, listed in Table AF18, calculate the accuracy of the clustering by flowClust, flowMerge and Misty Mountain (bottom of Table AF18).

**Table AF18 – Clusters assigned by different methods to the manually gated 4D OP9** data and the accuracy of the clustering methods

| Manual gating | Misty Mountain | flowClust | | flowMerge |
| --- | --- | --- | --- | --- |
|  |  | Optimal clust#:4 | Optimal clust#:8 | Optimal clust#7 |
| 1 | 5 | $\overline{4}$ | 2 | $\underline{3},\underline{4},\underline{5}$ |
| 2 | 2 | 2 | $\underline{1},\underline{3},\underline{8}$ | 1 |
| 3 | 3 | 1 | $\underline{4},\underline{6}$ | 7 |
| 4 | 1 | $\overline{4}$ | 7 | 6 |
| 5 | 4 | 3 | 5 | 2 |
| sensitivity | 5/5 | 3/5 | 3/5 | 4/5 |
| specificity | 5/5 | 3/4 | 3/8 | 4/7 |

(see legends to Table AF1)

**2.5 Clustering 5D simulated FCM data by different methods**

Model based clustering methods, such as flowClust, flowMerge and FLAME are designed to analyze data where the subpopulations follow certain distribution, while Misty Mountain’s performance is independent from the distribution. In this section we analyze simulated data where each of the five subpopulations follows a Gaussian distribution. This way we test Misty Mountain’s performance on a data set to which model based methods are designed for.

***2.5.1 Simulation of data***

The sum of 5 Gaussian distributions was simulated in 5D space (see Methods – main text). The 2D projections of the simulated 5D data are shown in Figure AF19.


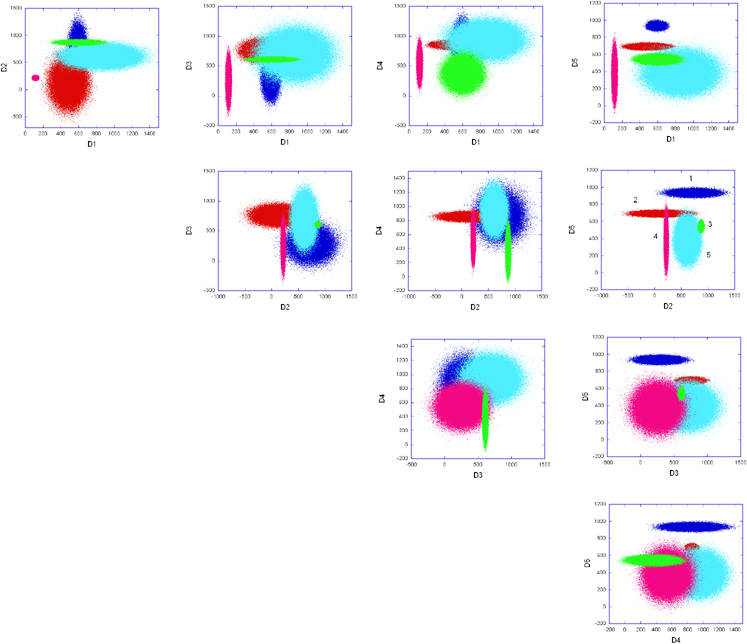


**Figure AF19. Five simulated 5D clusters.**

2D projections of the simulated 5D clusters. Each cluster follows a Gaussian distribution. The parameters of the Gaussians are listed in Table AF19. Code numbers of the clusters are: 1 (blue); 2 (red); 3 (green); 4(pink); 5(light blue).

The center coordinates, $X_{i}^{mean}$ and the standard deviations, $SD_{i}$ of each simulated Gaussian distribution were randomly generated within (0,1000) and (0,200) intervals, respectively (see Table AF19).

**Table AF19. Comparing cluster centers assigned by different**

***clustering methods to simulated 5D FCM data.***

| **Parameters of the simulated Gaussians** | | | | | |
| --- | --- | --- | --- | --- | --- |
|  | Gaussian #1 | Gaussian #2 | Gaussian #3 | Gaussian #4 | Gaussian #5 |
| Color code | blue | red | green | pink | light blue |
| # of data points | 100000 | 150000 | 200000 | 250000 | 300000 |
| $X_{1}^{mean}$ | 591.72 | 487.81 | 599.27 | 114.84 | 861.03 |
| $X_{2}^{mean}$ | 727.24 | 119.4 | 871.54 | 217.07 | 614.23 |
| $X_{3}^{mean}$ | 283.25 | 760.35 | 609.67 | 251.81 | 695.42 |
| $X_{4}^{mean}$ | 864.32 | 854.95 | 366.49 | 543.8 | 938.8 |
| $X_{5}^{mean}$ | 935.85 | 692.34 | 541.95 | 376.03 | 387.21 |
| $SD_{1}$ | 36.955 | 83.015 | 84.453 | 10.351 | 160.66 |
| $SD_{2}$ | 183.91 | 190.03 | 16.566 | 13.718 | 84.064 |
| $SD_{3}$ | 133.38 | 68.122 | 13.758 | 143.46 | 168.14 |
| $SD_{4}$ | 149.62 | 24.827 | 116.49 | 116.44 | 125.12 |
| $SD_{5}$ | 18.913 | 13.619 | 20.935 | 110.72 | 100.08 |
|  | | | | | |
| **Result of Misty Mountain clustering** | | | | | |
| Cluster code# | 4 | 3 | 1 | 2 | 5 |
| Cluster size, *C* | 65527 | 99632 | 1.44E+05 | 1.88E+05 | 7779 |
| $X_{1}^{center}$ | 593.97 | 487.67 | 599.62 | 114.66 | 861.78 |
| $X_{2}^{center}$ | 728.18 | 112.05 | 871.98 | 217.06 | 583.27 |
| $X_{3}^{center}$ | 281.79 | 759.73 | 610.36 | 252.66 | 698.9 |
| $X_{4}^{center}$ | 864.72 | 852.27 | 366.16 | 543.4 | 934.88 |
| $X_{5}^{center}$ | 937.53 | 691.64 | 542.15 | 376.09 | 387.35 |
|  | | | | | |
| **Result of flowClust clustering** | | | | | |
| Cluster code# | 3 | 1 | 5 | 4 | 2 |
| Cluster size, *C* | 99387 | 1.4917E+05 | 1.9889E+05 | 2.4846E+05 | 2.978E+05 |
| $X_{1}^{center}$ | 591.65 | 487.97 | 599.36 | 114.84 | 861.32 |
| $X_{2}^{center}$ | 727.29 | 119.68 | 871.52 | 217.07 | 614.11 |
| $X_{3}^{center}$ | 283.25 | 760.37 | 609.69 | 252.33 | 694.94 |
| $X_{4}^{center}$ | 864.83 | 855.03 | 366.72 | 543.64 | 938.91 |
| $X_{5}^{center}$ | 935.81 | 692.38 | 541.95 | 376.2 | 386.9 |
|  | | | | | |
| **Result of flowMerge clustering** | | | | | |
| Cluster code# | 3 | 4 | 2 | 5 | 1 |
| Cluster size, *C* | 91167 | 137042 | 182410 | 228396 | 273861 |
| $X_{1}^{center}$ | 591.67 | 487.81 | 599.37 | 114.83 | 861.11 |
| $X_{2}^{center}$ | 726.90 | 119.57 | 871.54 | 217.06 | 614.18 |
| $X_{3}^{center}$ | 283.40 | 760.36 | 609.68 | 252.21 | 695.05 |
| $X_{4}^{center}$ | 864.75 | 855.01 | 366.68 | 543.69 | 938.83 |
| $X_{5}^{center}$ | 935.80 | 692.37 | 541.92 | 376.33 | 387.02 |

In Table AF19 the center coordinates of each assigned cluster was calculated by averaging the i-th coordinates of the *C* cluster elements: $X_{i}^{center}=\sum_{j=1}^{C} X_{i}(j)/C$. The centers of the assigned clusters coincide quite well with the centers of the simulated Gaussians.

***2.5.2 Misty Mountain clustering***

When analyzing the 5D simulated data set Misty Mountain program randomly selected 600,000 points from the simulated 1 million points and ran the clustering on this reduced data set. The algorithm assigned 5 clusters to the simulated data within 196 sec. The analyzed histogram of the simulated data contained 11^5^ bins. The clusters contain 84% of the analyzed data points. Table AF20 lists the characteristics of the clusters assigned by Misty Mountain.

**Table AF20. Characteristics of the clusters assigned by**

**Misty Mountain to the 5D simulated FCM data**.

| Color code | Code # | $L_{p}$ | $L_{s}$ | $C$ | $f$ |
| --- | --- | --- | --- | --- | --- |
| Pink | 2 | 11579 | 90 | 188164 | 0.992 |
| Green | 1 | 27367 | 561 | 143646 | 0.980 |
| Red | 3 | 9560 | 561 | 99632 | 0.941 |
| Blue | 4 | 3477 | 117 | 65527 | 0.966 |
| Light blue | 5 | 732 | 423 | 7779 | 0.422 |

(see legends to Table 1 – main text)

The $f$ values in Table AF20 show that the simulated Gaussians are rather separated from each other except the light blue cluster that considerably overlaps with nearby cluster(s).

***2.5.3 Clustering by FLAME mixture model***

We attempted analyzing the 5D simulated data set by FLAME (version 4). We used again the default parameters and selected t-distribution. The analysis was run for cluster numbers from 3 to 8. The program ran more than 4 days when the job was removed from the queue.

***2.5.4 Clustering by flowClust mixture model***

Analyzing the 5D simulated data set by flowClust we used again the default parameters of the program. The analysis was run for cluster numbers from 1 to 10 and it took 10 hours 50 minutes. The optimal cluster number was selected by BIC and ICL. Table AF21 lists BIC and ICL for each cluster number. BIC levels off and ICL has maximum at cluster number 5. Thus according to these criterions the optimal cluster number is 5.

**Table AF21. BIC, ICL values calculated**

**at different cluster numbers by flowClust**

| Cluster number | BIC | ICL | Time in seconds |
| --- | --- | --- | --- |
| 1 | -6.83776E+07 | -6.83776E+07 | 171.051 |
| 2 | -6.61255E+07 | -6.61662E+07 | 1335.375 |
| 3 | -6.29854E+07 | -6.30264E+07 | 2399.496 |
| 4 | -5.99958E+07 | -5.99993E+07 | 2642.313 |
| 5 | -5.90300E+07 | -5.90336E+07 | 4904.268 |
| 6 | -5.90295E+07 | -5.90550E+07 | 3699.83 |
| 7 | -5.90289E+07 | -5.95934E+07 | 4334.21 |
| 8 | -5.90306E+07 | -5.92179E+07 | 4997.856 |
| 9 | -5.90288E+07 | -5.93275E+07 | 5772.076 |
| 10 | -5.90107E+07 | -5.95936E+07 | 8751.11 |
| optimal | 5 | 5 |  |

The centers of the 5 optimal clusters found by flowClust (listed in Table AF19) perfectly agree with the centers of the simulated Gaussians.

***2.5.5 Clustering by flowMerge mixture model***

Analyzing the 5D simulated data set by flowMerge we used the following parameters of the program: randomStart=50; B=1000; B.init=100; nu.est=1; tol.init=0.01; tol=1e-5. The analysis was run for cluster numbers from 1 to 10 and it took 31h 20min. The optimal cluster number, k=5, was selected by analyzing the entropy of clustering vs. the cumulative number of merged observations plot. In Table AF22 BIC, ICL, entropy, cumulative number and CPU time are listed for each cluster number.

**Table AF22. BIC, ICL, entropy and cumulative numbers calculated**

at different cluster numbers by flowMerge

| k | FlowClust BIC | FlowClust ICL | FlowMerge Entropy | Cumulative number | Time in seconds for flow.clust |
| --- | --- | --- | --- | --- | --- |
| 1 | -6.8E+07 | -6.8E+07 | 3.66E-11 | 0 | 766.338 |
| 2 | -6.6E+07 | -6.6E+07 | 0.176605 | 0.9E6 | 4233.675 |
| 3 | -6.3E+07 | -6.3E+07 | 14.82088 | 1.6E6 | 6371.689 |
| 4 | -6.1E+07 | -6.1E+07 | 103.4768 | 2.3E6 | 7885.776 |
| 5 | -5.9E+07 | -5.9E+07 | 791.4529 | 2.6E6 | 10353.03 |
| 6 | -5.9E+07 | -5.9E+07 | 544919.6 | 2.9 | 12242.14 |
| 7 | -5.9E+07 | -5.9E+07 | NA | NA | 14411.4 |
| 8 | -5.9E+07 | -5.9E+07 | NA | NA | 16604.09 |
| 9 | -5.9E+07 | -5.9E+07 | NA | NA | 18903.06 |
| 10 | -5.9E+07 | -5.9E+07 | NA | NA | 20976.77 |

The centers of the 5 optimal clusters found by flowMerge (listed in Table AF19) again perfectly agree with the centers of the simulated Gaussians.

As it was expected flowClust and flowMerge gave perfect clustering result. Misty Mountain also finds the five clusters but several order of magnitude faster than the model based methods. As a matter of fact FLAME was so slow that it was removed from the queue. Thus Misty Mountain is fast and accurate on analyzing both barcoding and simulated data sets for which model based clustering methods are not and are designed for, respectively.

**2.6 Clustering 2D simulated data by different methods. Case of non-convex shape cluster**

***2.6.1 Simulation of data***

State of the art model based clustering methods fit sum of convex shaped distributions to the FCM data. It is instructive to investigate what kind of cluster(s) are assigned to points that follow a non-convex shape distribution shown in Figure AF20a.

***2.6.2 Misty Mountain clustering***

Misty Mountain algorithm assigned 1 cluster to the 2D simulated distorted-Gaussian within 6 sec. The analyzed histogram of the simulated data contained 33^2^ bins. The clusters contain 100% of the analyzed data points. The assigned cluster is the same as in Figure AF20a.

**
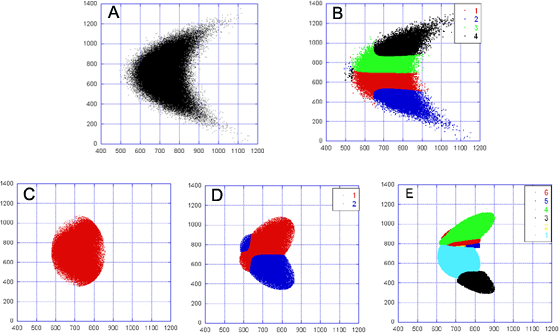
**

**Figure AF20. Simulated 2D non-convex shape cluster.** A) Distorted-Gaussian distribution simulated by using Monte Carlo techniques (see Methods – main text). Number of data points: 200,000, strength of distortion: s=0.002. B) Clusters assigned by FLAME to the 2D simulated distorted Gaussian. Case of optimal cluster number 4. Colored numbers in the upper right corner are the code numbers assigned by FLAME to each cluster. Points of similar color represent a cluster according to the FLAME clustering. C,D) Clusters assigned by flowClust to the 2D simulated distorted Gaussian. C) Case of optimal cluster number 1. D) Case of optimal cluster number 2. Colored numbers in the upper right corner are the code numbers assigned by flowClust to each cluster. Points of similar color represent a cluster according to the flowClust clustering. E) Clusters assigned by flowMerge to the 2D simulated distorted Gaussian. Optimal cluster number 6. Colored numbers in the upper right corner are the code numbers assigned by flowMerge to each cluster. Points of similar color represent a cluster according to the flowMerge clustering.

***2.6.3 Clustering by FLAME mixture model***

Analyzing the data set shown in Figure AF20a by FLAME (version 4) we used the default parameters and selected t-distribution. The analysis was run for cluster numbers from 1 to 4 and it took 2 hours and 49 minutes. The optimal cluster number was selected by BIC, AIC and SWR. Table AF23 lists the calculated BIC, AIC and SWR parameter values at each cluster number. Based on these parameter values an optimal cluster number was given by FLAME (see last row in Table AF23).

**Table AF23. SWR, BIC and AIC values calculated**

**at different cluster numbers by FLAME**

| Cluster number (k) | SWR | BIC | AIC |
| --- | --- | --- | --- |
| 1 | Inf | 4696467 | 4696406 |
| 2 | 0.905481 | 4652413 | 4652281 |
| 3 | 0.786287 | 4644754 | 4644550 |
| 4 | 0.687008 | 4643468 | 4643193 |
| optimal | 4 | 1 | 1 |

In the case of optimal cluster number 1 every point of the simulated distorted-Gaussian was assigned by FLAME to the single cluster, i.e.: the assigned cluster is the same as in Figure AF20a. In the case of optimal cluster number 4 the clusters assigned by FLAME are shown in Figure AF20b.

***2.6.4 Clustering by flowClust mixture model***

Analyzing the data shown in Figure AF20a by flowClust we used the default parameter values of the program. The analysis was run from cluster number 1 to 10. The optimal cluster number was selected by BIC and ICL. Table AF24 lists BIC and ICL for each cluster number. BIC levels off and ICL has maximum at cluster number 2 and 1, respectively, i.e. the two criterions result in different optimal cluster numbers, 2 and 1. The computation time for each cluster number is shown in the last column of Table AF24. The total run time was almost 2 hours.

**Table AF24. BIC, ICL values calculated**

**at different cluster numbers by flowClust**

| Cluster number (k) | BIC | ICL | Time in seconds |
| --- | --- | --- | --- |
| 1 | -4704517 | -4704517 | 11.028 |
| 2 | -4666032 | -4821309 | 128.592 |
| 3 | -4658046 | -4899339 | 249.661 |
| 4 | -4655724 | -4969749 | 406.056 |
| 5 | -4652418 | -5009244 | 574.447 |
| 6 | -4651422 | -5059289 | 731.334 |
| 7 | -4650066 | -5097691 | 1003.72 |
| 8 | -4649496 | -5145058 | 1148.496 |
| 9 | -4649282 | -5158409 | 1427.862 |
| 10 | -4648984 | -5211310 | 1309 |
| optimal | 2 | 1 |  |

In the case of optimal cluster numbers 1 and 2 the clusters assigned by flowClust are shown in Figure AF20c and AF20d, respectively.

***2.6.5 Clustering by flowMerge mixture model***

Analyzing the data shown in Figure AF20a by flowMerge we used the following parameters of the program: randomStart=50; B=1000; B.init=100; nu.est=1; tol.init=0.01; tol=1e-5. The analysis was run for cluster numbers from 1 to 10 and it took 1h 53min. The optimal cluster number, k=6, was selected by analyzing the entropy of clustering vs. the cumulative number of merged observations plot. In Table AF25 BIC, ICL, entropy, cumulative number and CPU time are listed for each cluster number.

**Table AF25. BIC, ICL, entropy and cumulative numbers calculated**

**at different cluster numbers by flowMerge**

| Cluster number (k) | Flowclust BIC | Flowclust ICL | Flowmerge Entropy | Cumulative number | Time in seconds for flow.clust |
| --- | --- | --- | --- | --- | --- |
| 1 | -4696236 | -4696236 | 4.68E-12 | 0.0E5 | 10.348 |
| 2 | -4651934 | -4806638 | 128347.6108 | 2.0E5 | 122.88 |
| 3 | -4644589 | -4892506 | 279097.2744 | 3.5E5 | 235.73 |
| 4 | -4644323 | -5012514 | 421735.3117 | 5.0E5 | 392.65 |
| 5 | -4644027 | -5094227 | 547837.8196 | 6.3E5 | 558.41 |
| 6 | -4643316 | -5151519 | 670790.0533 | 7.7E5 | 711.30 |
| 7 | -4643505 | -5209136 | 787591.4923 | 8.5E5 | 980.31 |
| 8 | -4643040 | -5257658 | 886706.3813 | 9.0E5 | 1122.99 |
| 9 | -4643068 | -5299539 | NA | NA | 1395.97 |
| 10 | -4643304 | -5337367 | NA | NA | 1280.60 |

The six clusters assigned by flowMerge are shown in Figure AF20e.

**2.7 Comparison of clustering methods. Concluding remarks**

Six sets of experimental and simulated FCM data were analyzed by five clustering methods: FLAME, flowClust, flowMerge, flowJo and Misty Mountain clustering. The comparison of the clustering results leads us to the following observations:

FlowJo and Misty Mountain are unsupervised clustering methods, identifying clusters in the FCM data without additional information. However, FLAME, flowClust and flowMerge require an interval of cluster numbers as input and select the optimal cluster number by using one of the available criterions.

Frequently different criterions result in different optimal cluster numbers. FLAME tends to suggest either the lower or the upper bound of the interval as the optimal cluster number even if the correct cluster number is inside the interval. Since these bounds are user defined the obtained cluster number is subjective.

flowClust usually suggests optimal cluster number within the interval. When analyzing manually gated 2D and 4D data the optimal cluster number did not agree with the correct number. Also the selection of the optimal cluster number is uncertain when it is defined by the location where the BIC function levels off or the considered function has more than one prominent maximum.

No one-to-one correspondence has been found between the manual gating of 2D and 4D data and the results of state of the art clustering methods: FLAME, flowClust, flowMerge and flowJo. These methods sometimes assigned one cluster to multiple gated clusters, sometimes assigned more than one clusters to one gated cluster. On the other hand Misty Mountain clustering was in agreement with the results of the manual gatings.

FLAME, flowClust and flowMerge are computationally very expensive clustering methods relative to flowJo or Misty Mountain. The result of the comprehensive comparison of the state of the art clustering methods, i.e. the accuracy and run time, is summarized in Table 3 (main text).

**References**

1. Jang W: **Nonparametric density estimation and clustering in astronomical sky survey**. *Comput Stat Data Anal* 2006, **50**:760-774.

2. Jang W, Hendry M: **Cluster analysis of massive datasets in astronomy**. *Statistics and Computing* 2007, **17**:253-262.

3. Cuevas A, Febrero M, Fraiman R: **Estimating the number of clusters**. *Can J Stat* 2000, **28**:367-382.

4. Cuevas A, Febrero M, Fraiman R: **Cluster analysis: a further approach based on density estimation**. *Comput Stat Data Anal* 2001, **36**:441-459.

5. Silverman BW: **Algorithm AS 176: Kernel density estimation using the fast Fourier transform.** *Applied Statistics* 1982, **31**:93-99.

6. Knuth KH: **Optimal data-based binning for histograms**. *arXiv:physics/0605197v1 [physicsdata-an]* 2006.

7. Hoshen J, Kopelman R: **Percolation and cluster distribution. 1. Cluster multiple labeling technique and critical concentration algorithm**. *Physical Review B* 1976, **14**(8):3438-3445.

8. Pyne S, Hu X, Wang K, Rossin E, Lin T-I, Mailer LM, Baecher-Allan C, McLachlan GJ, Tamayo P, Hafler DA *et al*: **Automated high dimensional flow cytometric data analysis**. *Proc Natl Acad Sci USA* 2009, **106**:8519-8524.

9. Lo K, Brinkman RR, Gottardo R: **Automated gating of flow cytometry data via robust model-based clustering.** *Cytometry* 2008, **73**:321-332.

10. Roederer M, Treister A, Moore W, Herzenberg LA: **Probability binning comparison: A metric for quantitating univariate distribution differences.** *Cytometry* 2001, **45**:37-46.

11. Roederer M, Moore W, Treister A, Hardy RR, Herzenberg LA: **Probability binning comparison: a metric for quantitating multivariate distribution differences.** *Cytometry* 2001, **45**:47-55.

12. Roederer M, Hardy RR: **Frequency difference gating: A multivariate method for identifying subsets that differ between samples.** *Cytometry* 2001, **45**:56-64.

13. Brinkman RR, Gasparetto M, Lee SJJ, Ribickas AJ, Perkins J, Janssen W, Smiley R, Smith C: **High-content flow cytometry and temporal data analysis for defining a cellular signature graft-versus-host disease**. *Biology of Blood and Marrow Transplantation* 2007, **13**(6):691-700.

14. Lo K, Hahne F, Brinkman RR, Gottardo R: **flowClust: a Bioconductor package for automated gating of flow cytometry data**. *Bmc Bioinformatics* 2009, **10**.
