# Supplementary material for: Misty Mountain clustering: application to fast unsupervised flow cytometry gating
Source: BMC Bioinformatics. 2010 Oct 9;11:502. doi: 10.1186/1471-2105-11-502 (PMC2967560; doi:10.1186/1471-2105-11-502)
Supplement: Additional file 2 — Table of cluster characteristics assigned to data in Figure 3a. (see legends to Table 1 - main text) The ith coordinate of the center of each cluster was calculated by averaging the ith coordinates of the C cluster elements: Xicenter=∑j=1CXi(j)/C [file 1471-2105-11-502-S2.DOC]

**Additional File 2 - Table of cluster** **characteristics assigned to data in Figure 3a**

| Code# | $L_{p}$ | $L_{s}$ | $C$ | $f$ | $X_{1}^{center}$ | $X_{2}^{center}$ |
| --- | --- | --- | --- | --- | --- | --- |
| 1 | 2065 | 151 | 51767 | 0.927 | 839.34 | 2565.3 |
| 2 | 3195 | 77 | 53457 | 0.976 | 1586.4 | 2591.9 |
| 3 | 3959 | 277 | 50048 | 0.93 | 2118.3 | 2559.9 |
| 4 | 3745 | 196 | 49251 | 0.948 | 2667.2 | 2552.3 |
| 5 | 4003 | 247 | 49842 | 0.938 | 3190 | 2564.1 |
| 6 | 1957 | 231 | 53805 | 0.882 | 842.66 | 1894.1 |
| 7 | 2494 | 277 | 52341 | 0.889 | 1594.1 | 1908.8 |
| 8 | 3361 | 237 | 50934 | 0.929 | 2121.5 | 1911.3 |
| 9 | 3490 | 247 | 50479 | 0.929 | 2687.8 | 1911.5 |
| 10 | 3454 | 186 | 48065 | 0.946 | 3196 | 1893.6 |
| 11 | 144 | 65 | 2585 | 0.549 | 758.24 | 1165.4 |
| 12 | 177 | 97 | 1794 | 0.452 | 1291.8 | 1221.6 |
| 13 | 193 | 117 | 1917 | 0.394 | 1813.3 | 1226.9 |
| 14 | 213 | 104 | 2124 | 0.512 | 2343 | 1183.7 |
| 15 | 207 | 114 | 2052 | 0.449 | 2876 | 1176.6 |
| 16 | 2933 | 595 | 49339 | 0.797 | 831.9 | 457.99 |
| 17 | 2908 | 727 | 43122 | 0.75 | 1521.7 | 456.07 |
| 18 | 3239 | 727 | 43148 | 0.776 | 2083.9 | 461.19 |
| 19 | 3406 | 613 | 45119 | 0.82 | 2652.5 | 461.61 |
| 20 | 3331 | 613 | 42920 | 0.816 | 3156.2 | 454.4 |
| 21 | 1 | 0 | 2 | 1 | 623.5 | 3359 |

(see legends to Table 1 – main text) The i^th^ coordinate of the center of each cluster was calculated by averaging the i^th^ coordinates of the *C* cluster elements: $X_{i}^{center}=\sum_{j=1}^{C} X_{i}(j)/C$
