# Supplementary figures and images for: Misty Mountain clustering: application to fast unsupervised flow cytometry gating
Source: BMC Bioinformatics. 2010 Oct 9;11:502. doi: 10.1186/1471-2105-11-502 (PMC2967560; doi:10.1186/1471-2105-11-502)

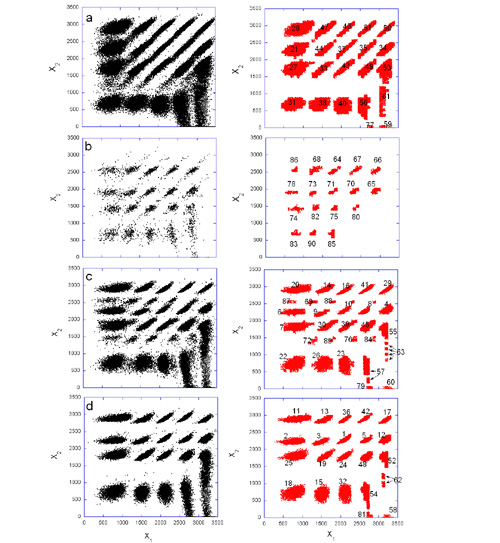

Supplement: Additional file 3 — Figure of three-dimensional FCM data. 853,674 U937 cells are stained by three florescence dyes, Pacific Blue, ALEXA-350-A and APC-Cy7-A. The fluorescence intensities of these dyes are plotted on the X1, X2 and X3 axes, respectively. By creating equidistant meshes of the X3 axis from the lowest to the highest intensity the three dimensional data space is divided into 46 slices. Left panels refer to the a) 6th, b) 16th, c) 26th and d) 35th slice of the data space. Right panels show the respective slices from the result of the cluster analysis. In the four slices all the assigned 89 clusters are represented. Each cluster is colored by red and marked by a code number. Code number 1 refers to the cluster containing the largest number of data points, number 2 to the second largest, etc. Virtually disconnected clusters with similar code number are in reality connected at a nearby slice. Table in Additional File 4 lists the characteristics of the resulting clusters. The optimal histogram contained 46 × 46 × 46 bins, and the cluster analysis required 11.2 seconds CPU time. [file 1471-2105-11-502-S3.TIFF]

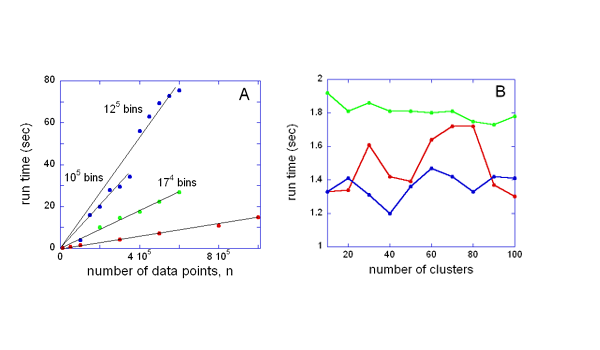

Supplement: Additional file 5 — Figures of simulation results on Misty Mountain clustering time complexity. Misty Mountain clustering has been performed on a series of simulated datasets. a) The run time of each analysis (dot) is plotted against the number of respective data points. Red dots: the datasets simulate the same 4 Gaussians in 2D (as in Figure 1a) but contain different number of points. Green dots: the datasets simulate the same 7 Gaussians in 4D but contain different number of points. Blue dots: the datasets simulate the same 5 Gaussians in 5D but contain different number of points. At a fixed bin number the run time increases linearly with the number of data points. b) The run time is plotted against the number of simulated Gaussians. In each of these simulations the number of data points were kept the same: 100,000, while the dimension of the data space was: 2D (red curve), 3D (blue curve), 5D (green curve). [file 1471-2105-11-502-S5.TIFF]
