# Supplementary material for: Misty Mountain clustering: application to fast unsupervised flow cytometry gating
Source: BMC Bioinformatics. 2010 Oct 9;11:502. doi: 10.1186/1471-2105-11-502 (PMC2967560; doi:10.1186/1471-2105-11-502)
Supplement: Additional file 4 — Table of cluster characteristics assigned to data in Additional File 3. (see legends to Table in Additional File 2) [file 1471-2105-11-502-S4.DOC]

**Additional File 4 - Table of cluster** **characteristics assigned to data in Additional File 3**

| Code# | $L_{p}$ | $L_{s}$ | $C$ | $f$ | $X_{1}^{center}$ | $X_{2}^{center}$ | $X_{3}^{center}$ |
| --- | --- | --- | --- | --- | --- | --- | --- |
| 1 | 1061 | 7 | 14754 | 0.993 | 2138.4 | 2317.3 | 2604.9 |
| 2 | 437 | 7 | 14489 | 0.984 | 831.65 | 2318.3 | 2648.6 |
| 3 | 875 | 9 | 14120 | 0.99 | 1581.2 | 2304.9 | 2645.1 |
| 4 | 922 | 11 | 14068 | 0.988 | 3200.9 | 2317.8 | 1910.7 |
| 5 | 1002 | 25 | 13958 | 0.975 | 2692.9 | 2305.5 | 2597.7 |
| 6 | 484 | 18 | 13522 | 0.963 | 840.46 | 2327.9 | 1998.8 |
| 7 | 338 | 20 | 13253 | 0.941 | 858.38 | 1785.6 | 1854 |
| 8 | 1076 | 34 | 13203 | 0.968 | 2690.6 | 2337.1 | 1973.2 |
| 9 | 851 | 47 | 13127 | 0.945 | 1603.8 | 2307.9 | 2010.9 |
| 10 | 940 | 31 | 13104 | 0.967 | 2135.3 | 2328.4 | 1959.1 |
| 11 | 486 | 13 | 13090 | 0.973 | 897.02 | 2906.5 | 2566.2 |
| 12 | 1009 | 22 | 13082 | 0.978 | 3178.9 | 2301.6 | 2569 |
| 13 | 774 | 12 | 13012 | 0.984 | 1604.6 | 2892.2 | 2592.6 |
| 14 | 684 | 12 | 12895 | 0.982 | 1611.9 | 2925.5 | 1908.4 |
| 15 | 380 | 7 | 12849 | 0.982 | 1580.5 | 726.97 | 2613.8 |
| 16 | 1019 | 17 | 12774 | 0.983 | 2142.3 | 2912.4 | 1949.3 |
| 17 | 1129 | 34 | 12757 | 0.97 | 3228.5 | 2913.7 | 2644 |
| 18 | 248 | 10 | 12659 | 0.96 | 775.55 | 716.97 | 2491.3 |
| 19 | 756 | 13 | 12505 | 0.983 | 1582.8 | 1773.9 | 2518.7 |
| 20 | 325 | 12 | 12459 | 0.963 | 899.44 | 2921.3 | 1908.2 |
| 21 | 284 | 28 | 12424 | 0.901 | 830.72 | 2292.5 | 458.74 |
| 22 | 204 | 15 | 12374 | 0.926 | 793.14 | 729.86 | 1841 |
| 23 | 287 | 17 | 12281 | 0.941 | 2082.1 | 702.47 | 1842.8 |
| 24 | 714 | 7 | 12277 | 0.99 | 2131.4 | 1815.6 | 2575.8 |
| 25 | 376 | 7 | 12225 | 0.981 | 851.18 | 1815.9 | 2555.6 |
| 26 | 275 | 17 | 12211 | 0.938 | 1543.2 | 732.51 | 1875.2 |
| 27 | 321 | 26 | 12170 | 0.919 | 815.84 | 1736.2 | 469.84 |
| 28 | 268 | 15 | 12083 | 0.944 | 914.85 | 2904 | 455.12 |
| 29 | 871 | 10 | 12075 | 0.989 | 3204.6 | 2904.1 | 1912.1 |
| 30 | 517 | 18 | 12071 | 0.965 | 1606.3 | 1792.8 | 1841.2 |
| 31 | 349 | 36 | 11961 | 0.897 | 753.24 | 689.24 | 439.54 |
| 32 | 323 | 12 | 11932 | 0.963 | 2070.6 | 691.3 | 2469.9 |
| 33 | 580 | 55 | 11929 | 0.905 | 1507.7 | 1728.9 | 470.33 |
| 34 | 614 | 51 | 11906 | 0.917 | 3158.1 | 2296.6 | 458.93 |
| 35 | 753 | 48 | 11859 | 0.936 | 2676.2 | 2315.1 | 461.03 |
| 36 | 1055 | 47 | 11685 | 0.955 | 2124.9 | 2888.2 | 2571.5 |
| 37 | 629 | 55 | 11679 | 0.913 | 2123.3 | 2315.1 | 461.65 |
| 38 | 340 | 36 | 11607 | 0.894 | 1420.8 | 685.3 | 443.17 |
| 39 | 531 | 25 | 11599 | 0.953 | 2125.6 | 1805.4 | 1901.8 |
| 40 | 319 | 30 | 11556 | 0.906 | 2031.1 | 641.27 | 444.75 |
| 41 | 961 | 11 | 11554 | 0.989 | 2692.9 | 2922.1 | 1920.8 |
| 42 | 860 | 31 | 11519 | 0.964 | 2651.5 | 2908.6 | 2576.2 |
| 43 | 524 | 41 | 11416 | 0.922 | 2062.8 | 1753.2 | 476.7 |
| 44 | 572 | 62 | 11346 | 0.892 | 1578.5 | 2300.4 | 459.36 |
| 45 | 571 | 22 | 11228 | 0.961 | 2698.4 | 1822.1 | 1909.6 |
| 46 | 600 | 62 | 11176 | 0.897 | 2130.9 | 2881.4 | 460.89 |
| 47 | 460 | 28 | 11110 | 0.939 | 1595.3 | 2904.7 | 459.83 |
| 48 | 635 | 11 | 11100 | 0.983 | 2681.7 | 1793.8 | 2557.8 |
| 49 | 493 | 51 | 10390 | 0.897 | 2652.6 | 1723.6 | 477.99 |
| 50 | 538 | 48 | 10276 | 0.911 | 3186.1 | 2886 | 463.35 |
| 51 | 678 | 47 | 9788 | 0.931 | 2679.7 | 2907.7 | 457.14 |
| 52 | 507 | 61 | 8366 | 0.88 | 3187.4 | 1779 | 2576.3 |
| 53 | 283 | 38 | 7892 | 0.866 | 3157 | 1737.8 | 457.12 |
| 54 | 200 | 52 | 7428 | 0.74 | 2614.3 | 673.27 | 2460.1 |
| 55 | 342 | 57 | 6551 | 0.833 | 3189.4 | 1778.8 | 1935.9 |
| 56 | 173 | 58 | 6429 | 0.665 | 2574.6 | 639.85 | 449.46 |
| 57 | 187 | 62 | 6068 | 0.668 | 2626.6 | 650.23 | 1836 |
| 58 | 532 | 38 | 4803 | 0.929 | 3206.2 | 4.631 | 2474.5 |
| 59 | 451 | 42 | 4706 | 0.907 | 3178 | 4.279 | 444.95 |
| 60 | 453 | 33 | 4494 | 0.927 | 3212.3 | 8.259 | 1810.5 |
| 61 | 81 | 42 | 1930 | 0.481 | 3076.8 | 804.31 | 444 |
| 62 | 121 | 61 | 1590 | 0.496 | 3093.7 | 968.36 | 2460.6 |
| 63 | 38 | 6 | 1260 | 0.842 | 1076.9 | 2552 | 1174.3 |
| 64 | 94 | 57 | 1162 | 0.394 | 3118.4 | 1085.8 | 1812.9 |
| 65 | 43 | 10 | 747 | 0.767 | 1798.4 | 2546 | 1204.7 |
| 66 | 46 | 9 | 716 | 0.804 | 2861.4 | 1943.7 | 1161.5 |
| 67 | 51 | 10 | 686 | 0.804 | 2868 | 2525 | 1163.2 |
| 68 | 47 | 10 | 665 | 0.787 | 2348.5 | 2549.4 | 1176.3 |
| 69 | 52 | 18 | 481 | 0.654 | 1273.8 | 2541.9 | 1916.8 |
| 70 | 55 | 18 | 476 | 0.673 | 2341.1 | 1951.5 | 1212.4 |
| 71 | 45 | 16 | 474 | 0.644 | 1826.2 | 1981.5 | 1240.6 |
| 72 | 39 | 13 | 432 | 0.667 | 1291.5 | 1405.8 | 1890.7 |
| 73 | 36 | 14 | 405 | 0.611 | 1308.6 | 1967.8 | 1292 |
| 74 | 19 | 7 | 394 | 0.632 | 791.63 | 1430.6 | 1193.5 |
| 75 | 31 | 15 | 380 | 0.516 | 1817.4 | 1464.2 | 1228.7 |
| 76 | 34 | 14 | 373 | 0.588 | 2339.2 | 1409.8 | 1859.3 |
| 77 | 90 | 58 | 368 | 0.356 | 2740.9 | 37.03 | 424.55 |
| 78 | 20 | 9 | 367 | 0.55 | 760.31 | 1963.8 | 1254.8 |
| 79 | 98 | 62 | 342 | 0.367 | 2774.5 | 19.161 | 1901.6 |
| 80 | 27 | 12 | 323 | 0.556 | 2390.1 | 1488.6 | 1219.5 |
| 81 | 87 | 52 | 323 | 0.402 | 2765.7 | 35.616 | 2541.7 |
| 82 | 29 | 12 | 317 | 0.586 | 1305.8 | 1445.1 | 1175.2 |
| 83 | 18 | 7 | 306 | 0.611 | 776.27 | 695.28 | 1141.3 |
| 84 | 32 | 10 | 283 | 0.688 | 2862.8 | 1455.5 | 1899.9 |
| 85 | 23 | 10 | 269 | 0.565 | 1754.6 | 699.44 | 1180.7 |
| 86 | 28 | 15 | 233 | 0.464 | 719.91 | 2520.7 | 1848.3 |
| 87 | 56 | 32 | 227 | 0.429 | 1798.3 | 2532.3 | 1889.5 |
| 88 | 44 | 22 | 202 | 0.5 | 1805.4 | 1455.7 | 1915.2 |
| 89 | 22 | 10 | 158 | 0.545 | 1269.2 | 700.67 | 1212.8 |

(see legends to Table in Additional File 2)
