# Supplementary material for: Misty Mountain clustering: application to fast unsupervised flow cytometry gating
Source: BMC Bioinformatics. 2010 Oct 9;11:502. doi: 10.1186/1471-2105-11-502 (PMC2967560; doi:10.1186/1471-2105-11-502)
Supplement: Additional file 9 — Table of the parameters of 8 distorted-Gaussian distributions simulated in 10D. The sum of 8 distorted-Gaussian distributions was simulated in 10D space with distortion parameter s = 0.004 (see Methods). The center coordinates, Ximean and the standard deviations, SDi of each distorted-Gaussian were randomly generated within (0,1000) and (0,200) intervals, respectively (see Methods). [file 1471-2105-11-502-S9.DOC]

**Additional File 9 - Table of parameters of 8 distorted-Gaussian**

**distributions simulated in 10D**

|  | Distorted  Gaussian #1 | Distorted  Gaussian #2 | Distorted  Gaussian #3 | Distorted  Gaussian #4 | Distorted  Gaussian #5 | Distorted  Gaussian #6 | Distorted  Gaussian #7 | Distorted  Gaussian #8 |
| --- | --- | --- | --- | --- | --- | --- | --- | --- |
| # of data points | 10000 | 11000 | 12000 | 13000 | 14000 | 15000 | 16000 | 9000 |
| $X_{1}^{mean}$ | 15.968 | 825.67 | 89.896 | 852.88 | 586.07 | 380.6 | 126 | 622.33 |
| $X_{2}^{mean}$ | 346.55 | 514.46 | 272.03 | 385.1 | 141.72 | 471.14 | 138.53 | 892.68 |
| $X_{3}^{mean}$ | 677.77 | 237.64 | 784.62 | 220.46 | 735.43 | 878.12 | 159.34 | 103.8 |
| $X_{4}^{mean}$ | 795.11 | 686.27 | 250.17 | 30.778 | 589.47 | 447.88 | 664.91 | 667.85 |
| $X_{5}^{mean}$ | 736.91 | 415.07 | 685.96 | 229.59 | 693.32 | 270.82 | 682.4 | 846.94 |
| $X_{6}^{mean}$ | 215.27 | 772.5 | 29.214 | 984.37 | 720.86 | 109.49 | 596.04 | 697.02 |
| $X_{7}^{mean}$ | 368.6 | 283.56 | 640.84 | 782.89 | 196.83 | 615.59 | 939.85 | 638.01 |
| $X_{8}^{mean}$ | 189.53 | 871.19 | 912.79 | 205.89 | 416.08 | 153.29 | 367.95 | 358.67 |
| $X_{9}^{mean}$ | 523.64 | 792.75 | 573.04 | 195.61 | 403.49 | 151.6 | 280.03 | 852.64 |
| $X_{10}^{mean}$ | 8.8771 | 834.33 | 655.01 | 400.93 | 218.38 | 83.488 | 113.09 | 311.72 |
| $SD_{1}$ | 127.18 | 149.11 | 3.7593 | 132 | 188.66 | 59.231 | 153.11 | 167.93 |
| $SD_{2}$ | 137.23 | 132.85 | 142.06 | 192.2 | 34.999 | 51.593 | 38.109 | 153.83 |
| $SD_{3}$ | 72.516 | 111.26 | 124.84 | 22.625 | 13.304 | 35.59 | 124.24 | 165.31 |
| $SD_{4}$ | 131.82 | 5.1529 | 162.25 | 170.38 | 156.71 | 68.236 | 152.99 | 198.01 |
| $SD_{5}$ | 32.247 | 86.118 | 56.159 | 175.8 | 6.8063 | 78.482 | 140.24 | 86.852 |
| $SD_{6}$ | 98.706 | 16.904 | 99.686 | 59.667 | 1.4763 | 149.53 | 167.62 | 48.871 |
| $SD_{7}$ | 22.896 | 196.27 | 100.59 | 69.767 | 163.18 | 173.68 | 7.9512 | 175.12 |
| $SD_{8}$ | 177.92 | 171.09 | 166.12 | 49.506 | 81.835 | 98.053 | 116.3 | 102.42 |
| $SD_{9}$ | 161.33 | 43.217 | 11.346 | 4.3787 | 39.35 | 72.792 | 119.09 | 135.99 |
| $SD_{10}$ | 98.947 | 37.383 | 193.04 | 29.155 | 160.89 | 125.32 | 167.71 | 128.38 |

The sum of 8 distorted-Gaussian distributions was simulated in 10D space with distortion parameter s=0.004 (see Methods). The center coordinates, $X_{i}^{mean}$ and the standard deviations, $SD_{i}$ of each distorted-Gaussian were randomly generated within (0,1000) and (0,200) intervals, respectively (see Methods).
