# Supplementary material for: Misty Mountain clustering: application to fast unsupervised flow cytometry gating
Source: BMC Bioinformatics. 2010 Oct 9;11:502. doi: 10.1186/1471-2105-11-502 (PMC2967560; doi:10.1186/1471-2105-11-502)
Supplement: Additional file 10 — Table of center coordinates of clusters assigned within the 5D subspace of the 10D simulated data. The Misty Mountain algorithm assigned 8 clusters to the 10D simulated data when the data were projected into a 5D subspace. The analyzed 5D histogram of the projected data contained 85 bins. The cluster elements were back-projected into the 10D data space. The clusters contain 72.7% of all the data points. The computation time was 9.4 sec. The Table lists the coordinates of each cluster center. The ith coordinate of the center of each cluster was calculated by averaging the ith coordinates of the C cluster elements: Xicenter=∑j=1CXi(j)/C. 8 out of the 8 cluster centers coincide with the centers of the 8 simulated distorted-Gaussians (listed in Table in Additional File 9). [file 1471-2105-11-502-S10.DOC]

**Additional File 10 - Table of center coordinates of clusters assigned within the 5D**

**subspace of the 10D simulated data**

| Cluster Code# | 1 | 2 | 3 | 4 | 5 | 6 | 7 | 8 |
| --- | --- | --- | --- | --- | --- | --- | --- | --- |
| $C$ | 11714 | 13436 | 11428 | 9819 | 6413 | 9756 | 7133 | 3018 |
| $X_{1}^{center}$ | 590.52 | 380.69 | 123.42 | 863.48 | 11.742 | 89.816 | 813.01 | 613.16 |
| $X_{2}^{center}$ | 143.54 | 470.04 | 138.53 | 391.76 | 350.59 | 275.9 | 506.35 | 898.86 |
| $X_{3}^{center}$ | 735.39 | 878.23 | 159.7 | 220.38 | 679.8 | 785.14 | 243.26 | 109.41 |
| $X_{4}^{center}$ | 592.78 | 448.61 | 662.84 | 31.281 | 796.34 | 253.02 | 686.25 | 691.46 |
| $X_{5}^{center}$ | 693.37 | 270.98 | 680.58 | 227.09 | 737.08 | 685.61 | 414.61 | 850.52 |
| $X_{6}^{center}$ | 720.87 | 112.39 | 598.04 | 985.92 | 208.53 | 28.798 | 772.1 | 695.36 |
| $X_{7}^{center}$ | 197.51 | 617.31 | 939.95 | 780.17 | 368.3 | 639.1 | 288.94 | 623.56 |
| $X_{8}^{center}$ | 414.97 | 155.45 | 366.9 | 208.89 | 178.67 | 909.48 | 874.36 | 360.67 |
| $X_{9}^{center}$ | 404.44 | 150.18 | 278.78 | 195.65 | 522.95 | 573.22 | 792.91 | 858.92 |
| $X_{10}^{center}$ | 219.74 | 83.039 | 114.52 | 401.53 | 5.209 | 655.74 | 834.45 | 310.34 |
| Coinciding with | Distorted  Gaussian #5 | Distorted  Gaussian #6 | Distorted  Gaussian #7 | Distorted  Gaussian #4 | Distorted  Gaussian #1 | Distorted  Gaussian #3 | Distorted  Gaussian #2 | Distorted  Gaussian #8 |

The Misty Mountain algorithm assigned 8 clusters to the 10D simulated data when the data were projected into a 5D subspace. The analyzed 5D histogram of the projected data contained 8^5^ bins. The cluster elements were back-projected into the 10D data space. The clusters contain 72.7% of all the data points. The computation time was 9.4 sec. The Table lists the coordinates of each cluster center. The i^th^ coordinate of the center of each cluster was calculated by averaging the i^th^ coordinates of the *C* cluster elements: $X_{i}^{center}=\sum_{j=1}^{C} X_{i}(j)/C$. 8 out of the 8 cluster centers coincide with the centers of the 8 simulated distorted-Gaussians (listed in Table in Additional File 9).
